# Herbal Medicine Intervention for the Treatment of COVID-19: A Living Systematic Review and Cumulative Meta-Analysis

**DOI:** 10.3389/fphar.2022.906764

**Published:** 2022-06-20

**Authors:** Lin Ang, Eunhye Song, Xiao-Yang Hu, Hye Won Lee, Yaolong Chen, Myeong Soo Lee

**Affiliations:** ^1^ KM Science Research Division, Korea Institute of Oriental Medicine, Daejeon, South Korea; ^2^ Global Cooperation Center, Korea Institute of Oriental Medicine, Daejeon, South Korea; ^3^ School of Primary Care, Population Sciences and Medical Education, Aldermoor Health Centre, University of Southampton, Southampton, United Kingdom; ^4^ KM Convergence Research Division, Korea Institute of Oriental Medicine, Daejeon, South Korea; ^5^ Evidence Based Medicine Center, School of Basic Medical Sciences, Lanzhou University, Lanzhou, China; ^6^ WHO Collaborating Centre for Guideline Implementation and Knowledge Translation, Lanzhou University, Lanzhou, China; ^7^ Korean Convergence Medicine, University of Science and Technology, Daejeon, South Korea

**Keywords:** COVID-19 evidence, efficacy, integrative herbal medicine, living review, safety

## Abstract

**Background:** Integrative herbal medicine has been reported to have beneficial effects in the treatment of coronavirus disease 2019 (COVID-19).

**Aim:** To compile up-to-date evidence of the benefits and risks of herbal medicine for the treatment of COVID-19 symptoms.

**Methods:** Eleven databases, including PubMed, Cochrane Register of Controlled Trials (CENTRAL), Embase, Allied and Complementary Medicine Database (AMED), Chinese National Knowledge Infrastructure Database (CNKI), Wanfang Database, and Chinese Science and Technique Journals Database (VIP), Research Information Service System (RISS), Korean Medical database (KMBase), Korean Association of Medical Journal database (KoreaMed), and OASIS database, were searched from 15 June, 2020, until 28 March 2022. Randomized controlled trials (RCTs), published in any language, reporting the efficacy and safety outcomes of herbal medicine in patients of all ages with a PCR-confirmed diagnosis of COVID-19 were included in this analysis. Data extraction and quality assessments were performed independently.

**Results:** Random-effects meta-analyses showed evidence of favorable effects of treatment with herbal medicine when added to standard treatment, versus standard treatment alone, on the total effective rate (*p* = 0.0001), time to remission from fever (*p* < 0.00001), rate of remission from coughing (*p* < 0.0001), fatigue (*p* = 0.02), sputum production (*p* = 0.004), improvement of manifestations observed on chest computed tomography scans (*p* < 0.00001), incidence of progression to severe COVID-19 (*p* = 0.003), all-cause mortality (*p* = 0.003), time to a negative COVID-19 coronavirus test (*p* < 0.0001), and duration of hospital stay (*p* = 0.0003). There was no evidence of a difference between herbal medicine added to standard treatment, versus standard treatment alone, on the rate of remission from symptoms such as a fever, sore throat, nasal congestion and discharge, diarrhea, dry throat, chills, and the rate of conversion to a negative COVID-19 coronavirus test. Meta-analysis showed no evidence of a significant difference in adverse events between the two groups. There was an unclear risk of bias across the RCTs included in this analysis, indicating that most studies had methodological limitations.

**Conclusion:** Current evidence suggests that herbal medicine added to standard treatment has potential benefits in the treatment of COVID-19 symptoms but the certainty of evidence was low.

## Introduction

Coronavirus disease 2019 (COVID-19), also known as severe acute respiratory syndrome coronavirus 2 (SARS-CoV-2), has caused a significant impact on global health. Although the number of new cases is ebbing in several countries that were hit hard early on, the number of new cases worldwide is growing rapidly each day. As of 25 March 2022, COVID-19 has afflicted more than 476 million people globally with approximately 6.1 million fatalities (World Health Organization (WHO), 2022b). This pandemic has a broad clinical spectrum ranging from self-limiting respiratory tract illness to fatal pneumonia, with therapeutic measures remaining limited (Cao et al., 2020).

Being the first country heavily affected by the COVID-19 outbreak, the Chinese government has attributed its swift turnaround in managing COVID-19 to the integration of herbal medicine with conventional medicine (National Health Commission of the People's Republic of China, 2020; Weeks, 2020). Clinical practice guidelines or memorandums of herbal medicine usage for COVID-19 have also been issued in several countries such as South Korea, Japan, Malaysia, and India.

Compelling evidence supporting integrative herbal medicine have been reported in several review articles amongst numerous studies published following the outbreak of COVID-19. A recent systematic review reported that the integration of herbal medicine and conventional medicine can alleviate the symptoms of COVID-19 without increasing adverse drug reactions (Liu M. et al., 2020). However, such beneficial effects of herbal medicine are inconclusive due to the poor quality of the methodology used in the clinical trials. Another review article critically analyzed the therapeutic evidence of herbal medicine integration in the management of COVID-19 symptoms and concluded that, by combining with conventional medicine, herbal medicine showed positive effects and great potential in complementing the conventional treatment (Chan et al., 2020). Yet, the evidence is not sufficient enough and more clinical studies are required to validate this conclusion. Overall, the evidence has shown a level of uncertainty in the effectiveness of herbal medicine intervention for the treatment of COVID-19, and rigorous clinical studies are highly warranted.

To address the uncertainty in the evidence for herbal medicine intervention for COVID-19 treatment, there is a need for the continuous surveillance of new evidence. This can be performed using a living systematic review (LSR) approach, where a systematic review is continually updated to incorporate newly available evidence (Elliott et al., 2017). According to the International Clinical Trials Registry Platform (ICTRP) of the WHO, >100 randomized controlled trials (RCTs) related to herbal medicine intervention have been registered worldwide, and there is the probability of more trials in the near future [World Health Organization (WHO), 2022a]. Hence, there is likely to be emerging evidence that will allow informed decisions to be made on the effectiveness of treatment using herbal medicine for the treatment of COVID-19 symptoms.

In this LSR, evidence regarding the potential effects and risks of using herbal medicine for the treatment or prevention of COVID-19 was evaluated, focusing on the following questions: *1*) Is herbal medicine effective in treating patients with COVID-19? *2*) Is herbal medicine effective in preventing COVID-19 infections? *3*) Are there any potential adverse events or risks associated with the use of herbal medicine for the treatment of COVID-19? New evidence will be kept under surveillance and summarized quarterly. A major update will be performed if the emerging evidence impacts upon the conclusion of this review. This LSR will continuously inform the best practice for clinical treatment and clinical research of this disease.

## Methods

This LSR was registered in the PROSPERO international prospective register of systematic reviews database (ID: CRD42020191711) and was reported in accordance with the reporting guideline provided in the Preferred Reporting Items Systematic Reviews and Meta-Analysis (PRISMA) statement (Moher et al., 2009). Standard methods and guidance for conducting living reviews was also followed (Akl et al., 2017; Elliott et al., 2017).

### Data Sources and Searches

All sources were searched from the date of inception through to 28 March 2022. Two review authors developed the search strategy (*see* Supplementary Table S1) and conducted the search monthly for eligible articles. The sources included, but was not limited to the following 11 databases: PubMed, Cochrane Register of Controlled Trials (CENTRAL), Embase, Allied and Complementary Medicine Database (AMED), Chinese National Knowledge Infrastructure Database (CNKI), Wanfang Database, and Chinese Science and Technique Journals Database (VIP), Research Information Service System (RISS), Korean Medical database (KMBase), Korean Association of Medical Journal database (KoreaMed), and OASIS database.

Additional sources such as the COVID-19 Study Registry (https://covid-19.cochrane.org/), WHO’s COVID-19 Database (https://search.bvsalud.org/global-literature-on-novel-coronavirus-2019-ncov/), and The Institute of Medical Information (IMI) and Library, Chinese Academy of Medical Sciences and Peking Union Medical College (CAMS and PUMC) (http://2019ncov.imicams.ac.cn/index.html) were also searched. To identify ongoing trials, the National Institute of Health and Clinical Trials Database (http://www.clinicaltrials.gov/), WHO’s International Clinical Trials Registry Platform (https://www.who.int/ictrp/en/), and Chinese Clinical Trial Registry (http://www.chictr.org.cn/) were searched.

There were no restrictions concerning the language or publication status. The use of indexing terms such as medical subject headings (MeSH) terms, and other equivalent terms, were applied for wider coverage. All of the searches will be performed again on a monthly basis to retrieve any further eligible studies.

### Eligibility Criteria

#### Types of Studies

All RCTs that included herbal medicine as a treatment for COVID-19 were eligible for inclusion. Other clinical and experimental studies such as case-control studies, cohort studies, cross-sectional studies, case reports, animal studies, and laboratory experiments were excluded. Preprint articles were also excluded because these studies can introduce bias and could undergo major changes before peer-review and publication.

#### Types of Participants

Patients of all ages with a PCR-confirmed diagnosis of COVID-19, regardless of sex, or ethnicity were included. Patients who used herbal medicine for other comorbidities or any other purpose were excluded to ensure the accuracy of evidence. Participants who were suspected of having COVID-19 or asymptomatic PCR-confirmed COVID-19 cases were also excluded. Trials which reported separate data on symptomatic and asymptomatic COVID-19 participants were included in this review.

#### Types of Interventions

All types of herbal medicine intervention by oral administration, such as herbal decoctions or a patient’s medicine were considered in this review, irrespective of the composition of herbal medicine, dose, and duration of administration. Co-intervention of standard medical treatment was also eligible so long as the co-intervention was given similarly in both intervention and comparison groups. Herbal medicine interventions in the form of single/multiple herbal extractions, herbal injections, herbal fumigation, or combinations of two or more different types of herbal medicine interventions were excluded.

#### Types of Comparisons

Comparison groups that received only standard medical treatment, placebo, or no treatment were included. Comparator groups that involved any type of herbal medicine were excluded.

#### Outcome Measures

The primary outcomes comprised of:

− Total effective rate (defined by the number of patients who were cured or where treatment was markedly effective)
− Symptom resolution

The secondary outcomes included:

− Chest radiological findings
− Progression to severe or critical COVID-19
− All-cause mortality
− Negative viral assay
− Duration of hospital stay
− Adverse events

Both primary and secondary outcomes were chosen by referring to the core outcome sets (COS) that have been registered on the Core Outcome Measures in Effectiveness Trials (COMET) database (Qiu R. et al., 2020; Jin et al., 2020; Marshall et al., 2020; Tong et al., 2020), with the consideration of applicability to patients with mild-to-critical COVID-19.

### Study Selection

Two review authors (LA and ES) performed the literature searches and assessed the eligibility of the studies. To prevent discrepancies between daily database updates, all searches were conducted and results exported on the same day. The full text of the potentially eligible RCTs was then retrieved and screened according to the pre-defined criteria. Any discrepancies regarding the suitability of a study for inclusion in this review were discussed with a third review author (ML) until a consensus was reached.

### Data Extraction and Quality Assessment

Trial design, sample size, risk of bias domains (as defined above), length of trial and follow-up, the number of participants (recruited, randomized, withdraw, completed, analyzed, and lost to follow-up), age range, sex ratio, details of the interventions and comparators (regimens), all outcome measures, study results, adverse events, and funding source data were extracted by two review authors (LA and ES) independently using a standard data extraction form. Each trial was named after the first author and year of primary publication, and any relevant secondary publications were classified under that name. The authors of the included studies were contacted for unreported data or missing data.

Subsequently, two review authors (LA and ES) individually assessed the risk of bias of the included studies using the Cochrane Collaboration’s Risk of Bias Assessment tool Version 2 (ROB 2.0) (Sterne et al., 2019). The following five domains were assessed: *1*) randomization process, *2*) deviations from intended interventions, *3*) missing outcome data, *4*) measurement of outcome, and *5*) selection of the reported results. The risk of bias of each item was categorized into “low risk of bias,” “some concerns,” or “high risk of bias.” The overall risk of bias of the included studies was also assessed. Additionally, the quality of evidence was summarized using the Grading of Recommendations, Assessment, Development, and Evaluations (GRADE, https://gradepro.org/). Any disagreements over the data extraction, risk of bias, and quality of evidence in a particular study were resolved through the involvement of a third party.

### Data Synthesis and Analysis

All data were analyzed using Review Manager (RevMan) Version 5.3 software. The risk ratios (RRs), odds ratio (OR), or risk difference (RD) with 95% confidence intervals (CIs) were calculated for dichotomous data while the mean differences (MDs) with 95% CIs were calculated for continuous data. A narrative synthesis of the key findings from the included studies was presented according to the review questions with summary tables for study characteristics, participants, and outcome details. Quantitative synthesis was also performed to demonstrate overall effect estimates. The random-effects model was used as it incorporates heterogeneity both within and between studies. The heterogeneity levels of the eligible RCTs were assessed using I^2^ statistics.

### Living Review

A monthly surveillance for new evidence related to the potential benefits and risks of the treatment was planned. The data selection, data extraction, and quality assessment methods described in this review will be performed. The relevant meta-analyses will continuously be updated, and if significant evidence is available, the results will be published quarterly.

## Results

### Study Characteristics

**FIGURE 1:**
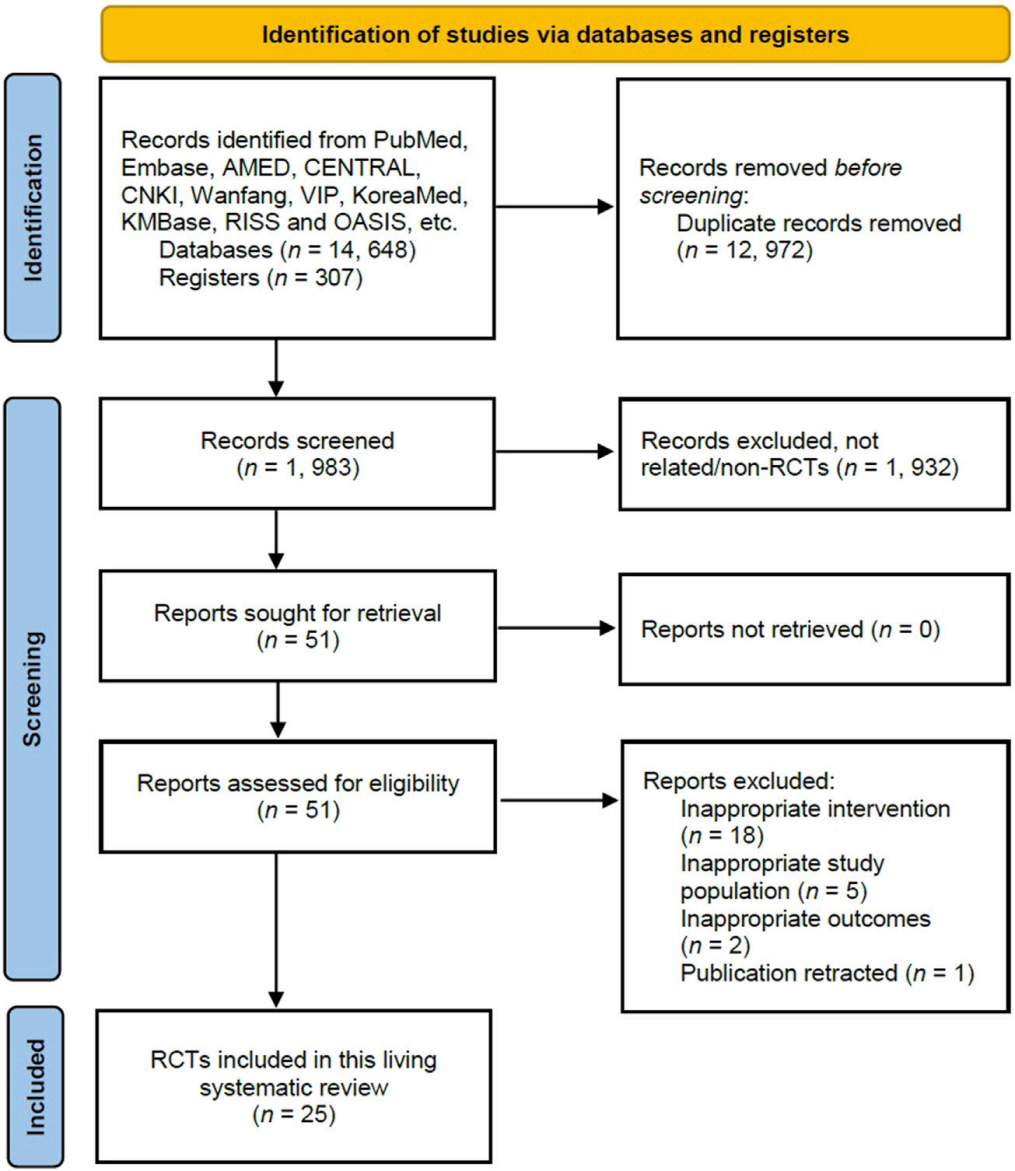
Study selection process.

Following screening of 14,955 titles and abstracts, and removing duplicates from the literature searches, 51 full-text records were retrieved. A total of 25 randomized clinical trials (2,288 participants) were identified as of 28 March 2022 (Figure 1) and included in this review.(Ai et al., 2020; Ding et al., 2020; Duan et al., 2020; Fu et al., 2020a; Fu et al., 2020b; Hu et al., 2020; Li and Zhang, 2020; Liao, 2020b; a; Lin et al., 2020; Liu Z. et al., 2020; Qiu M. et al., 2020; Sun H.-m. et al., 2020; Wang JB. et al., 2020; Wang Y. et al., 2020; Xiong WZ. et al., 2020; G-CHAMPS Collaborative Group, 2020; Yu et al., 2020; Zhang C. et al., 2020; Zhang Y. et al., 2020; Zhao et al., 2020; Zheng et al., 2020; Liu J. et al., 2021; Zeng et al., 2021; Zhou et al., 2021) Reasons for exclusion of 26 full-text articles are provided in Supplementary Table S2. Most trials included in this review were not registered (18/25; 72%), published in Chinese (16/25; 64%), and evaluated treatment in symptomatic COVID-19 patients (2,272/2,288; 99%). All studies included in this analysis were conducted in China.

The diagnosis of COVID-19 was confirmed by polymerase chain reaction (PCR) for all cases in all studies and was accompanied by radiological findings of pneumonia caused by COVID-19 in some studies. The majority of standard medical treatment received by participants involved the use of lopinavir-ritonavir, arbidol, alpha-interferon nebulization, or a combination of these treatments. Five different forms of herbal medicines were evaluated. The most common form of herbal medicines was herbal decoction (12/25; 48%), followed by herbal granules (9/25; 36%), herbal oral liquid (2/25; 8%), herbal capsules (1/25; 4%), and herbal powder (1/25; 4%). All trials compared herbal medicine added to standard treatment versus standard treatment alone. Due to insufficient data, meta-analysis for several trials and outcomes could not be conducted. We wrote to the authors of those studies requesting the missing data and have yet to receive responses. The detailed study characteristics of the included trials can be found in Tables 1, 2.

**TABLE 1:** Study characteristics of trials conducted with herbal medicine for the treatment of COVID-19.

Study, Year (Ref)	Sample size (N, M/F) Age (years)	Severity of Course of disease (days)	Study groups	Comparator	Outcome measures	Trial registration Country
Ding 2020 (Ding et al., 2020)	100 (78/22) 54.7/50.8	Mild to severe 5.3/6.0	(A) HM (Qingfei Touxie Fuzheng decoction, two times daily for 10 days, *n* = 51), plus B	(B) ST (α-interferon nebulization + 0.9% sodium chloride injection + ribavirin 500 mg intravenous drip + Quinolone antibacterial drugs and/or 3rd generation cephalosporins, *n* = 49)	*1*) Rate of complete remission from clinical symptoms *2*) Rate of improvement for chest CT *3*) Adverse events	No information China
Li 2020 (Li and Zhang, 2020)	12 (7/5) 52.0/50.0	Severe n.r.	(A) HM (Qingfei Paidu decoction, two times daily for 6 days, *n* = 6), plus B	(B) ST (supportive treatment such as oxygen therapy, antiviral medications and symptomatic therapies, *n* = 6)	*1*) Total effective rate *2*) Duration of hospital stay *3*) Adverse events	No information China
Liao (a) 2020 (Liao, 2020b)	70 (37/33) 60.3/63.2	Mild to severe n.r.	(A) HM (Self-prescribed decoction, once daily, *n* = 35), plus B	(B) ST (lopinavir/ritonavir for two times daily, *n* = 35)	*1*) Rate of improvement for clinical symptoms *2*) Rate of recovery of chest CT manifestations *3*) Adverse events	No information China
Liao (b) 2020 (Liao, 2020a)	70 (38/32) 65.3/67.2	n.r.	(A) HM (Self-prescribed decoction, once daily, *n* = 35), plus B	(B) ST (lopinavir/ritonavir for two times daily, *n* = 35)	*1*) Rate of improvement for clinical symptoms *2*) Adverse events	No information China
Lin 2020 (Lin et al., 2020)	82 (38/44) 46.0/43.8	Moderate 5.1/6.0	(A) HM (Xuanfei Qingre decoction, two times daily for 7 days, *n* = 6), plus B	(B) ST (α-interferon nebulization, 50 μg + lopinavir/ritonavir, 200 mg + 50 mg, twice daily, *n* = 24)	*1*) Total effective rate *2*) Time to complete remission from clinical symptoms *3*) Rate of recovery of chest CT manifestations *4*) Conversion rate to a negative viral assay *5*) Progression to severe COVID-19 *6*) Duration of hospital stay *7*) Adverse events	No information China
Qiu 2020 (Qiu M. et al., 2020)	50 (27/23) 53.4/51.3	Moderate 2.8/3.2	(A) HM (Maxing Xuanfei Jiedu decoction, three times daily for 10 days, *n* = 25), plus B	(B) ST (α-interferon nebulization + Kaletra/Aluvia (Lopinavir/Ritonavir), two times daily, *n* = 25)	*1*) Time to complete remission from clinical symptoms *2*) Rate of recovery of chest CT manifestations *3*) Progression to severe COVID-19	No information China
Xiong 2020 (Xiong et al., 2020a)	42 (n.r.) 57.1/62.4	Mild to severe n.r.	(A) HM (Xuanfei Baidu decoction, two times daily for 7 days, *n* = 22), plus B	(B) ST (Lopinavir/Ritonavir, 200 mg for two times daily, *n* = 20)	*1*) Rate of complete remission from clinical symptoms *2*) Adverse events	ChiCTR2000034795 China
Ye 2020 (G-CHAMPS Collaborative Group, 2020)	42 (7/35) 65.0/59.0	Severe 9.0/9.5	(A) HM (Herbal decoction^a^, two times daily for 7 days, *n* = 28), plus B	(B) ST (Lopinavir/Ritonavir, 200 mg for two times daily, *n* = 14)	*1*) Total effective rate *2*) Mortality	ChiCTR2000029418 China
Zhao 2020 (Zhao et al., 2020)	39 (22/17) n.r.	n.r.	(A) HM (Yidu-toxicity blocking lung decoction, *n* = 15), plus B	(B) ST (supportive treatment such as oxygen therapy, antiviral medications and symptomatic therapies, *n* = 24)	*1*) Total effective rate *2*) Duration of hospital stay	No information China
Zheng 2020 (Zheng et al., 2020)	130 (86/44) 17-84/18-85	Moderate to severe n.r.	(A) HM (Xiao Chaihu plus Maxing Shigan Decoction/Sanren decoction, three times daily for 14 days, *n* = 65), plus B	(B) ST (α-interferon nebulization + lopinavir and ritonavir tablets + arbidol tablets + Moxifloxacin + Whey protein powder + Methylprednisolone, *n* = 65)	*1*) Total effective rate	No information China
Zeng 2021 (Zeng et al., 2021)	59 (40/19) 53.3/50.7	Mild to moderate n.r.	(A) HM (Maxingshigan-Weijing decoction, two times daily for 14 days, *n* = 29), plus B	(B) ST (supportive treatment such as oxygen therapy, antiviral medications and symptomatic therapies, *n* = 30)	*1*) Rate of complete remission from clinical symptoms *2*) Time to remission from clinical symptoms *3*) Progression to severe COVID-19 *4*) Conversion time to a negative viral assay *5*) Adverse events	ChiCTRC2000030759 China
Zhang 2020 (b) (Zhang C. et al., 2020)	45 (19/26) 53.7/55.6	Moderate n.r.	(A) HM (Jiawei Dayuan Yin, three times daily for 7 days, *n* = 22), plus B	(B) ST (Lopinavir/Ritonavir, 200 mg for two times daily, *n* = 23)	*1*) Time to remission from clinical symptoms *2*) Rate of recovery of chest CT manifestations *3*) Adverse events	No information China
Ai 2020 (Ai et al., 2020)	98 (41/57) 44.0/45.0	Mild to severe 4.6/5.3	(A) HM (Feiyan Yihao granules, two times daily for 12 days, *n* = 55) plus B	(B) ST (Arbidol or lopinavir/ritonavir or hydroxychloroquine + supportive treatment such as oxygen therapy, antiviral medications and symptomatic therapies, *n* = 43)	*1*) Total effective rate *2*) Duration of hospital stay *3*) Adverse events	No information China
Duan 2020 (Duan et al., 2020)	123 (62/51) 52.0/50.3	Mild 2.7/2.5	(A) HM (Jinhua Qinggan granules, three times daily for 5 days, *n* = 82), plus B	(B) ST (Lopinavir/ Ritonavir, 200 mg + Chloroquine Phosphate tablets, 500 mg + Alpha interferon and ribavirin injection for two times daily + Arbidol Hydrochloride tablets, 500 mg for three times daily, *n* = 41)	*1*) Rate of complete remission from clinical symptoms *2*) Adverse events	No information China
Fu (a) 2020 (Fu et al., 2020a)	73 (38/35) 45.3/44.7	Moderate 7.6/8.5	(A) HM (Toujie Quwen granules, four times daily for 15 days, *n* = 37), plus B	(B) ST (Arbidol Hydrochloride Tablets, 0.2 g per time, three times daily + Ambroxol Hydrochloride Tablets, 30 mg three times daily, *n* = 36)	*1*) Total effective rate *2*) Adverse events	No information China
Fu (b) 2020 (Fu et al., 2020b)	65 (36/29) 43.3/43.7	Mild to moderate 7.6/8.5	(A) HM (Toujie Quwen granules, two times daily for 10 days, *n* = 32), plus B	(B) ST (Arbidol Hydrochloride Tablets, 200 mg + Moxifloxacin, 400 mg + Ambroxol Hydrochloride Tablets, 30 mg for three times daily, *n* = 33)	*1*) Total effective rate *2*) Rate of improvement for chest CT *3*) Progression to severe COVID-19 *4*) Adverse events	No information China
Liu 2020 (Liu Z. et al., 2020)	80 (37/43) 50.73/51.75	Moderate to severe >24 h	(A) HM (Jinhua Qinggan granules, two times daily for 7 days, *n* = 44), plus B	(B) ST (supportive treatment such as oxygen inhalation, and symptomatic and supportive treatment, *n* = 36)	*1*) Time to a negative viral assay conversion *2*) Adverse events	No information China
Sun 2020 (Sun H.-m. et al., 2020)	57 (28/29) 45.4/42.0	Mild to moderate 11.7/13.0	(A) HM (Lianhua Qingke granules, three times daily for 14 days, *n* = 32), plus B	(B) ST (Lopinavir/Ritonavir + Alpha interferon injection, two times daily, *n* = 25)	*1*) Rate of complete remission from clinical symptoms *2*) Rate of improvement for chest CT *3*) Progression to severe COVID-19	No information China
Yu 2020 (Yu et al., 2020)	295 (171/124) 48.2/47.2	Mild to moderate n.r.	(A) HM (Lianhua Qingwen granules, two times daily for 7 days, *n* = 147), plus B	(B) ST (Arbidol Hydrochloride Tablets, 200 mg + Ambroxol Hydrochloride Tablets, 30 mg for three times daily + Moxifloxacin, 400 mg for one time daily, *n* = 148)	*1*) Total effective rate *2*) Rate of improvement for chest CT *3*) Progression to severe COVID-19 *4*) Adverse events	No information China
Zhou 2020 (Zhou et al., 2021)	111 (71/40) 66-total	Severe to critical 19/16	(A) HM (Shenhuang granules, two times daily, *n* = 61), plus B	(B) ST (supportive treatment such as oxygen therapy, antiviral medications and symptomatic therapies, *n* = 61)	*1*) Total effective rate *2*) Progression to severe COVID-19 *3*) Mortality *4*) Adverse events	ChiCTR2000029777 China
Liu 2021 (Liu M. et al., 2021)	195 (73/122) 56/56.5	Mild to severe n.r.	(A) HM (Huashi Baidu granules, two times daily for 14 days, *n* = 99), plus B	(B) ST (supportive treatment such as oxygen therapy, antiviral medications and symptomatic therapies, *n* = 96)	*1*) Rate of complete remission from clinical symptoms *2*) Time to remission from clinical symptoms *3*) Rate of recovery of chest CT manifestations *4*) Conversion time to a negative viral assay *5*) Adverse events	ChiCTRC2000030288 China
Hu 2020 (Hu et al., 2020)	284 (150/134) 50.4/51.8	n.r. 9.5/9.9	(A) HM (Lianhua Qingwen capsules, three times daily for 14 days, *n* = 142), plus B	(B) ST (supportive treatment such as oxygen therapy, antiviral medications and symptomatic therapies, *n* = 142)	*1*) Total effective rate *2*) Rate of complete remission from clinical symptoms *3*) Time to complete remission from clinical symptoms *4*) Rate of recovery of chest CT manifestations *5*) Conversion rate to a negative viral assay *6*) Time to a negative viral assay conversion *7*) Progression to severe COVID-19 *8*) Adverse events	ChiCTRC2000029434 China
Wang (a) 2020 (Wang J. B. et al., 2020)	48 (26/21) 46.8/51.4	n.r. 6.5/8.0	(A) HM (Keguan-1 powder, two times daily, *n* = 24), plus B	(B) ST (α-interferon nebulization, 50 μg + lopinavir/ritonavir, 400 and 100 mg for two times daily, *n* = 23)	*1*) Time to remission from clinical symptoms *2*) Rate of recovery of chest CT manifestations *3*) Conversion time to a negative viral assay *4*) Mortality *5*) Adverse events	NCT 04251871 China
Wang (b) 2020 (Wang Y. et al., 2020)	38 (20/18) 43.4/41.7	Moderate 6.5/5.6	(A) HM (Qingre Kangdu oral liquid, three times daily for 10 days, *n* = 11), plus B (C) Asymptomatic (HM, *n* = 8)	(B) ST (α-interferon nebulization, twice daily + Arbidol Hydrochloride Tablets, 200 mg three times daily, *n* = 11) (D) Asymptomatic (SC, *n* = 8)	*1*) Rate of complete remission from clinical symptoms *2*) Time to remission from clinical symptoms *3*) Rate of recovery of chest CT manifestations	No information China
Zhang 2020 (a) (Zhang Y. et al., 2020)	80 (73/47) 53.4/52.0	Moderate 2.7/2.4	(A) HM (Jinyinhua oral liquid, three times daily for 10 days, *n* = 80), plus B	(B) ST (Lopinavir/ritonavir tablets + Intramuscular injection of alpha-interferon, two times daily, *n* = 40)	*1*) Rate of complete remission from clinical symptoms *2*) Conversion rate to a negative viral assay *3*) Progression to severe COVID-19	No information China

CT, computed tomography; HM, herbal medicine; ST, standard treatment.

a Herbal decoction prescribed based on Guidelines for the Diagnosis and Treatment of 2019-nCoV by the National Health Commission of China.

**TABLE 2:** Compositions and usage of herbal medicine for the treatment of COVID-19.

Study, Year (Ref)	Formulation	Composition of prescriptions	Preparations	Usage of prescription	Quality control
Ding 2020 (Ding et al., 2020)	Qingfei Touxie Fuzheng decoction	*Ephedra sinica* Stapf 6 g, Calcium sulfate dihydrate 20 g, *Prunus armeniaca* L. 10 g, *Lonicera japonica* Thunb. 30 g, *Forsythia suspensa* (Thunb.) Vahl 15 g, *Phragmites australis* (Cav.) Trin. ex Steud. 30 g, *Coix* *lacryma-jobi* var. ma-yuen (Rom.Caill.) Stapf 30 g, *Bombyx mori* L. 10 g, *Cryptotympana pustulata* Fabricius 10 g, *Reynoutria japonica* Houtt. 15 g, *Curcuma longa* L. 10 g, *Paeonia lactiflora* Pall. 10 g, *Pseudostellaria heterophylla* (Miq.) Pax 20 g, *Glycyrrhiza glabra* L. 15 g	decoction	bid	n.r.
Li 2020 (Li and Zhang, 2020)	Qingfei Paidu decoction	*Ephedra sinica* Stapf 9 g, *Glycyrrhiza glabra* L. 6 g, *Prunus armeniaca* L. 9 g, Calcium sulfate dihydrate 15–30 g, *Neolitsea cassia* (L.) Kosterm. 9 g, *Alisma plantago-aquatica* subsp. *orientale* (Sam.) Sam. 9 g, *Crotalaria albida* B.Heyne ex Roth 9 g, *Atractylodes macrocephala* Koidz. 9 g, *Poria cocos* (Schw.) Wolf. 15 g, *Bupleurum falcatum* L. 16 g, *Scutellaria baicalensis* Georgi 6 g, *Pinellia ternata* (Thunb.) Makino 9 g, *Zingiber officinale* Roscoe 9 g, *Aster tataricus* L.f. 9 g, *Tussilago farfara* L. 9 g, *Iris domestica* (L.) Goldblatt & Mabb. 9 g, *Asarum heterotropoides* F.Schmidt 6 g, *Dioscorea oppositifolia* L. 12 g, *Citrus sinensis* Osbeck 6 g, *Citrus × aurantium* L. 6 g, *Pogostemon cablin* (Blanco) Benth. 9 g	decoction	bid	n.r.
Liao (a) 2020 (Liao, 2020b)	Self-prescribed decoction	**Mild stage**: *Magnolia officinalis* Rehder & E.H.Wilson 10 g, *Pogostemon cablin* (Blanco) Benth. 10 g, *Hansenia weberbaueriana* (Fedde ex H.Wolff) Pimenov & Kljuykov 10 g, *Zingiber officinale* Roscoe 10 g, *Areca catechu* L. 10 g, *Atractylodes lancea* (Thunb.) DC. 10 g, *Lanxangia tsao-ko* (Crevost & Lemarié) M.F.Newman & Skornick. 6 g, *Ephedra sinica* Stapf 6 g, *Atractylodes lancea* (Thunb.) DC. 15 g **Moderate stage**: *Prunus armeniaca* L. 10 g, *Descurainia sophia* (L.) Webb ex Prantl 10 g, *Prunus persica* (L.) Batsch 10 g, *Areca catechu* L. 10 g, Calcium sulfate dihydrate 30 g, *Trichosanthes kirilowii* Maxim. 30 g, *Ephedra sinica* Stapf 12 g, *Lanxangia tsao-ko* (Crevost & Lemarié) M.F.Newman & Skornick. 6 g, *Rheum palmatum* L. 6 g **Severe stage**: *Panax ginseng* C.A.Mey. 15 g, *Aconitum carmichaeli* Debeaux 10 g, *Cornus officinalis* Siebold & Zucc. 15 g	decoction	qd	n.r.
Liao (b) 2020 (Liao, 2020a)	Self-prescribed decoction	*Prunus armeniaca* L. 10 g, Calcium sulfate dihydrate 30 g, *Rheum palmatum* L. 6 g, *Prunus persica* (L.) Batsch 10 g, *Atractylodes lancea* (Thunb.) DC. 10 g, *Glycyrrhiza glabra* L. 4 g	decoction	qd	n.r.
Lin 2020 (Lin et al., 2020)	Xuanfei Qingre decoction	*Ephedra sinica* Stapf 9 g, *Prunus armeniaca* L. 12 g, Calcium sulfate dihydrate 30 g, *Glycyrrhiza glabra* L. 6 g, *Prunus persica* (L.) Batsch 12 g, *Benincasa hispida* (Thunb.) Cogn. 30 g, *Phragmites australis* (Cav.) Trin. ex Steud. 30 g, *Platycodon grandiflorus* (Jacq.) A. DC. 9 g, *Pinellia ternata* (Thunb.) Makino 12 g, *Allium chinense* G.Don 12 g, *Lanxangia tsao-ko* (Crevost & Lemarié) M.F.Newman & Skornick. 6 g, *Pogostemon cablin* (Blanco) Benth. 10 g	decoction	bid	n.r.
Qiu 2020 (Qiu M. et al., 2020)	Maxing Xuanfei Jiedu decoction	*Ephedra sinica* Stapf 9 g, *Prunus armeniaca* L. 12 g, Calcium sulfate dihydrate 15–30 g, *Fritillaria thunbergii* Miq. 12 g, *Cryptotympana pustulata* Fabricius 10 g, *Bombyx mori* L. 15 g, *Curcuma longa* L. 12 g, *Platycodon grandiflorus* (Jacq.) A. DC. 12 g, *Citrus x aurantium* L. or *Citrus sinensis* (L.) 12 g, *Lanxangia tsao-ko* (Crevost & Lemarié) M.F.Newman & Skornick. 9 g, *Wurfbainia compacta* (Sol. ex Maton) Skornick. & A.D.Poulsen 12 g	decoction	tid	n.r.
Xiong 2020 (Xiong W. Z. et al., 2020)	Xuanfei Baidu decoction	*Ephedra sinica* Stapf 8 g, *Prunus armeniaca* L. 15 g, Calcium sulfate dihydrate 30 g, *Atractylodes lancea* (Thunb.) DC. 10 g, *Coix lacryma-jobi* var. ma-yuen (Rom.Caill.) Stapf 30 g, *Pogostemon cablin* (Blanco) Benth. 15 g, *Reynoutria japonica* Houtt. 20 g, *Descurainia sophia* (L.) Webb ex Prantl 15 g, *Verbena officinalis* L. 30 g, *Phragmites australis* (Cav.) Trin. ex Steud. 30 g, *Artemisia annua* L. 25 g, *Citrus maxima* (Burm.) Merr. 20 g, *Glycyrrhiza glabra* L. 10 g	decoction	bid	n.r.
Ye 2020 (G-CHAMPS Collaborative Group, 2020)	Herbal decoction	Per the NHC-NATCM-China guideline	decoction	bid	In accordance with 2015 Chinese Pharmacopeia
Zhao 2020 (Zhao et al., 2020)	Yidu-toxicity blocking lung decoction	*Prunus armeniaca* L. 10 g, Calcium sulfate dihydrate 30 g, *Trichosanthes kirilowii* Maxim. 30 g, *Rheum palmatum* L. 6 g, *Ephedra sinica* Stapf 6 g, *Lepidium apetalum* Willd 10 g, *Prunus persica* (L.) Batsch 10 g, *Lanxangia tsao-ko* (Crevost & Lemarié) M.F.Newman & Skornick. 6 g, *Areca catechu* L. 10 g, *Atractylodes lancea* (Thunb.) DC. 10 g	decoction	n.r.	Manufactured by Guangdong E-fong Pharmaceutical. Drugs (License no 20160214)
Zheng 2020 (Zheng et al., 2020)	Xiao Chaihu plus Maxing Shigan Decoction/Sanren decoction	*Bupleurum falcatum* L. 20 g, *Scutellaria baicalensis* Georgi 12 g, *Pinellia ternata* (Thunb.) Makino 12 g, *Codonopsis pilosula* (Franch.) Nannf. 15 g, *Zingiber officinale* Roscoe 10 g, *Ziziphus jujuba* Mill. 12 g, *Glycyrrhiza glabra* L. 10 g, *Ephedra sinica* Stapf 10 g, *Prunus armeniaca* L. 12 g, Calcium sulfate dihydrate 30 g, *Phragmites australis* (Cav.) Trin. ex Steud. 30 g, *Aster tataricus* L.f. 15 g, *Tussilago farfara* L. 15 g, *Cryptotympana pustulata* Fabricius 10 g, *Coix lacryma-jobi* var. ma-yuen (Rom.Caill.) Stapf 20 g, *Hordeum vulgare* L. 20 g/ *Prunus armeniaca* L. 10 g, *Wurfbainia compacta* (Sol. ex Maton) Skornick. & A.D.Poulsen 10 g, *Coix lacryma-jobi* var. ma-yuen (Rom.Caill.) Stapf 30 g, *Magnolia officinalis* Rehder & E.H.Wilson 10 g, *Pinellia ternata* (Thunb.) Makino 10 g, *Tetrapanacis *Medulla** 10 g, *Glycyrrhiza glabra* L. 10 g, Hydrated magnesium silicate 10 g, *Anemarrhena asphodeloides* Bunge 10 g, *Scutellaria baicalensis* Georgi 10 g, *Ephedra sinica* Stapf 8 g, *Poria cocos* (Schw.) Wolf. 10 g, *Bupleurum falcatum* L. 15 g, *Lophatherum gracile* Brongn. 10 g	decoction	tid	n.r.
Zeng 2021 (Zeng et al., 2021)	Maxingshigan-Weijing decoction	*Ephedra sinica* Stapf 10 g, *Prunus armeniaca* L. 10 g, Calcium sulfate dihydrate 45 g, *Phragmites australis* (Cav.) Trin. ex Steud. 30 g, *Prunus persica* (L.) Batsch 20 g, *Benincasa hispida* (Thunb.) Cogn. 20 g, *Trichosanthes kirilowii* Maxim. 30 g, *Citrus × aurantium* L. 12 g, *Pinellia ternata* (Thunb.) Makino 12 g, *Bambusae Caulis* in Taenias 12 g, *Descurainia sophia* (L.) Webb ex Prantl 30 g, *Acorus calamus* var. angustatus Besser 15 g *Curcuma longa* L. 10 g, *Glycyrrhiza glabra* L. 5 g	decoction	bid	n.r.
Zhang 2020 (b) (Zhang C. et al., 2020)	Jiawei Dayuan Yin	*Ephedra sinica* Stapf 10 g, *Prunus armeniaca* L. 15 g, Calcium sulfate dihydrate 20 g, *Trichosanthes kirilowii* Maxim. 20 g, *Rheum palmatum* L. 6 g, *Descurainia sophia* (L.) Webb ex Prantl 10 g, *Prunus persica* (L.) Batsch 10 g, *Lanxangia tsao-ko* (Crevost & Lemarié) M.F.Newman & Skornick. 6 g, *Areca catechu* L. 10 g, *Atractylodes lancea* (Thunb.) DC. 10 g	decoction	tid	Manufactured by Xinlvse Pharmaceutical Industry Technology Development Co., Ltd.
Ai 2020 (Ai et al., 2020)	Feiyan Yihao granules	*Artemisia annua* L., *Astragalus mongholicus* Bunge, *Cremastra appendiculata*, *Pleione bulbocodioides*, *Forsythia suspensa* (Thunb.) Vahl, *Scutellaria baicalensis* Georgi, *Lonicera japonica* Thunb., *Isatis tinctoria* subsp. tinctoria, *Bupleurum falcatum* L., *Cryptotympana pustulata* Fabricius, *Kitagawia praeruptora* (Dunn) Pimenov, *Fritillaria cirrhosa* D.Don, *Fritillaria thunbergii* Miq., *Prunus mume* (Siebold) Siebold & Zucc., *Scrophularia ningpoensis* Hemsl., *Poria cocos* (Schw.) Wolf., *Pseudostellaria heterophylla* (Miq.) Pax	granules	bid	n.r.
Duan 2020 (Duan et al., 2020)	Jinhua Qinggan granules	*Lonicera japonica* Thunb., Calcium sulfate dihydrate, *Ephedra sinica* Stapf, *Prunus armeniaca* L., *Scutellaria baicalensis* Georgi, *Forsythia suspensa* (Thunb.) Vahl, *Fritillaria thunbergii* Miq., *Anemarrhena asphodeloides* Bunge, *Arctium lappa* L., *Artemisia annua* L., *Mentha canadensis* L., *Glycyrrhiza glabra* L.	granules	tid	Patented medicine: Z20160001
Fu (a) 2020 (Fu et al., 2020a)	Toujie Quwen granules	*Forsythia suspensa* (Thunb.) Vahl 30 g, *Cremastra appendiculata*, *Pleione bulbocodioides* 20 g, *Lonicera japonica* Thunb. 15 g, *Scutellaria baicalensis* Georgi 10 g, *Isatis tinctoria* subsp. tinctoria 10 g, *Bupleurum falcatum* L. 5 g, *Artemisia annua* L. 10 g, *Cryptotympana pustulata* Fabricius 10 g, *Kitagawia praeruptora* (Dunn) Pimenov 5 g, *Fritillaria cirrhosa* D.Don 10 g, *Fritillaria thunbergii* Miq. 10 g, *Prunus mume* (Siebold) Siebold & Zucc. 30 g, *Scrophularia ningpoensis* Hemsl. 10 g, *Astragalus mongholicus* Bunge 45 g, *Poria cocos* (Schw.) Wolf. 30 g, *Pseudostellaria heterophylla* (Miq.) Pax 15 g	granules	qid	Manufactured by Guangdong E-fong Pharmaceutical. Drugs
Fu (b) 2020 (Fu et al., 2020b)	Toujie Quwen granules	*Forsythia suspensa* (Thunb.) Vahl 30 g, *Cremastra appendiculata*, *Pleione bulbocodioides* 20 g, *Lonicera japonica* Thunb. 15 g, *Scutellaria baicalensis* Georgi 10 g, *Isatis tinctoria* subsp. tinctoria 10 g, *Bupleurum falcatum* L. 5 g, *Artemisia annua* L. 10 g, *Cryptotympana pustulata* Fabricius 10 g, *Kitagawia praeruptora* (Dunn) Pimenov 5 g, *Fritillaria cirrhosa* D.Don 10 g, *Fritillaria thunbergii* Miq. 10 g, *Poria cocos* (Schw.) Wolf. 30 g, *Prunus mume* (Siebold) Siebold & Zucc. 30 g, *Scrophularia ningpoensis* Hemsl. 10 g, *Astragalus mongholicus* Bunge 45 g, *Pseudostellaria heterophylla* (Miq.) Pax 15 g	granules	bid	Manufactured by Guangdong E-fong Pharmaceutical. Drugs (License no 20160214)
Liu 2020 (Liu Z. et al., 2020)	Jinhua Qinggan granules	n.r.	granules	bid	Patented medicine: Z20160001
Sun 2020 (Sun C. Y. et al., 2020)	Lianhua Qingke granules	*Ephedra sinica* Stapf, *Morus alba* L., *Prunus armeniaca* L., *Forsythia suspensa* (Thunb.) Vahl, *Lonicera japonica* Thunb., *Rheum palmatum* L. *etc*.	granules	tid	Manufactured by Shijiazhuang Yiling Pharmaceutical Co., Ltd (License no 2020LCKY-003)
Yu 2020 (Yu et al., 2020)	Lianhua Qingwen granules	n.r.	granules	bid	Patented medicine: Z20100040
Zhou 2020 (Zhou et al., 2021)	Shenhuang granules	*Panax ginseng* C.A.Mey. 50 g, *Rheum palmatum* L. 40 g, *Sargentodoxa cuneata* (Oliv.) Rehd. et Wils. 30 g, *Taraxaci Herba* 30 g, *Aconitum carmichaeli* Debeaux 50 g, *Hirudo nipponica* Whitman 6 g	granules	bid	Manufactured by Beijing Tcmages Pharmaceutical Co., Ltd. Approved by the China National Medical Product Administration (Approval number: Jing 20180032)
Liu 2021 (Liu J. et al., 2021)	Huashi Baidu granules	*Ephedra sinica* Stapf, *Prunus armeniaca* L., Calcium sulfate dihydrate, *Glycyrrhiza glabra* L., *Pogostemon cablin* (Blanco) Benth., *Magnolia officinalis* Rehder & E.H.Wilson, *Atractylodes lancea* (Thunb.) DC., *Lanxangia tsao-ko* (Crevost & Lemarié) M.F.Newman & Skornick., *Pinellia ternata* (Thunb.) *Makino Praeparatum*, *Poria cocos* (Schw.) Wolf., *Rheum palmatum* L., *Astragalus mongholicus* Bunge, *Descurainia sophia* (L.) Webb ex Prantl, *Paeonia lactiflora* Pall.	granules	bid	Manufactured by Huayi Pharmaceutical Co., Ltd.
Hu 2020 (Hu et al., 2020)	Lianhua Qingwen capsules	*Forsythia suspensa* (Thunb.) Vahl, *Lonicera japonica* Thunb., *Ephedra sinica* Stapf, *Isatis tinctoria* L., *Pogostemon cablin* (Blanco) Benth., *Rheum palmatum* L., *Glycyrrhiza glabra* L., *Dryopteris crassirhizoma* Nakai, *Rhodiola crenulata* (Hook.f. & Thomson) H.Ohba, *Houttuynia cordata* Thunb., *Prunus armeniaca* L., Calcium sulfate dihydrate, 1-menthol	capsules	tid	In accordance with The Pharmacopedia of People’s Republic of China UPLC analysis reported
Wang (a) 2020 (Wang et al., 2020a)	Keguan-1 powder	*Lonicera japonica* Thunb. 30 g, *Forsythia suspensa* (Thunb.) Vahl 30 g, *Morus alba* L. 15 g, *Chrysanthemum × morifolium* (Ramat.) Hemsl. 10 g, *Coix lacryma-jobi* var. ma-yuen (Rom.Caill.) Stapf 30 g, *Fritillaria thunbergii* Miq. 15 g, *Prunus armeniaca* L. 9 g	powder	bid	HPLC-MS analysis reported
Wang (b) 2020 (Wang Y. et al., 2020)	Qingre Kangdu oral liquid	n.r.	oral liquid	tid	n.r.
Zhang 2020 (a) (Zhang Y. et al., 2020)	Jinyinhua oral liquid	n.r.	oral liquid	tid	Manufactured by Zhenao Jinyinhua Drug Co.,Ltd.

bid, bis in die (twice daily); n.r., not reported; qd, quaque die (once daily); qid, quater in die (four times daily); tid, ter in die (three times daily).

### Risk of Bias and Certainty of Evidence

Most of the studies assessed had a concerning level of risk of bias. Of the RCTs included in this review, 16 trials performed adequate randomization using a simple randomization method (Ai et al., 2020; Qiu M. et al., 2020; Wang JB. et al., 2020; Xiong WZ. et al., 2020; Fu et al., 2020b; Sun H.-m. et al., 2020; Wang Y. et al., 2020; Ding et al., 2020; Duan et al., 2020; Hu et al., 2020; Lin et al., 2020; G-CHAMPS Collaborative Group, 2020; Yu et al., 2020; Liu J. et al., 2021; Zeng et al., 2021; Zhou et al., 2021). The specific information on the generation of randomization was absent in nine trials. Most of the trials did not report on allocation sequence concealment. Six trials were open-labeled (Sun H.-m. et al., 2020; Wang Y. et al., 2020; Hu et al., 2020; G-CHAMPS Collaborative Group, 2020; Liu J. et al., 2021; Zeng et al., 2021), one was double-blinded (Wang JB. et al., 2020), and the remaining trials did not provide information on masking. Of the seven trials with publicly accessible protocols or registrations, two trials did not report results for one or more outcomes that were pre-specified in their protocols (Hu et al., 2020; Zhou et al., 2021). The reporting bias in two trials was assessed as concerning due to selective reporting and missing outcome data (Zhao et al., 2020; Zeng et al., 2021). One trial was initially posted as a preprint and subsequently peer-reviewed and published, and no substantial discrepancies in reporting were determined between the preprint and journal publication (G-CHAMPS Collaborative Group, 2020). The full risk of bias assessments for each domain for each study and the summary of findings are available in Supplementary Tables S3, S4, and Figure 1.

### Therapeutic Effect of Herbal Medicine

#### Total Effective Rate

**FIGURE 2:**
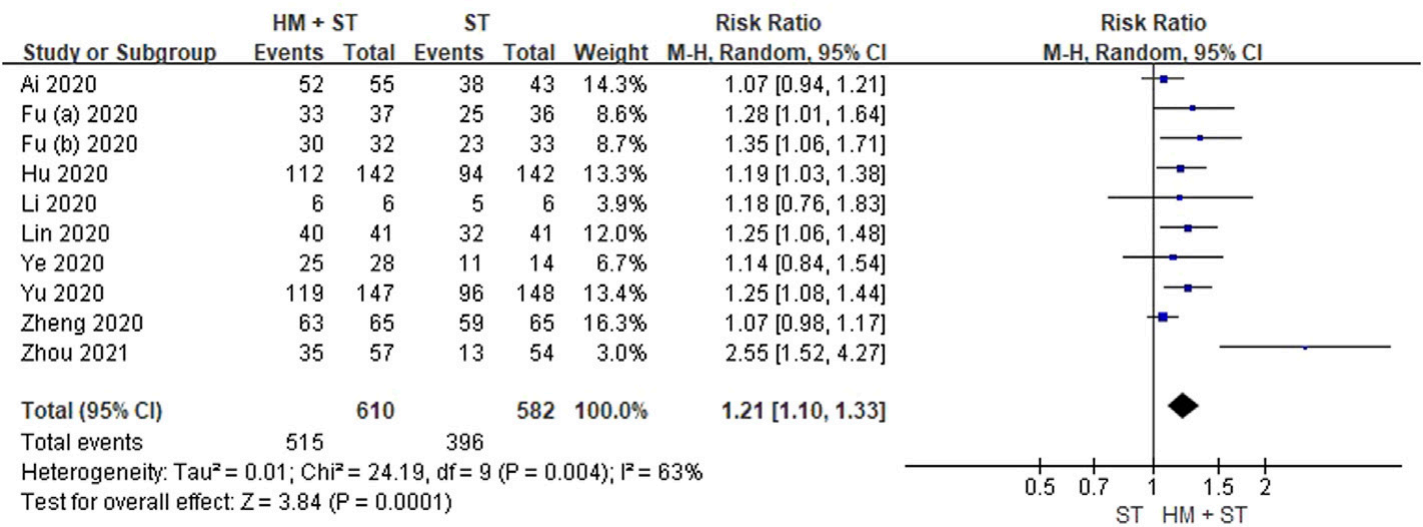
Forest plot for total effective rate of herbal medicine treatment in COVID-19 patients.

Ten RCTs including 1,192 participants, reported total effective rates. Meta-analysis showed that herbal medicine added to standard treatment was more favorable compared with standard treatment alone for total effective rate (RR 1.21, 95% CI 1.10 to1.33, *p* = 0.0001, I^2^ = 63; low certainty, Figure 2).

#### Symptom Resolution

**FIGURE 3:**
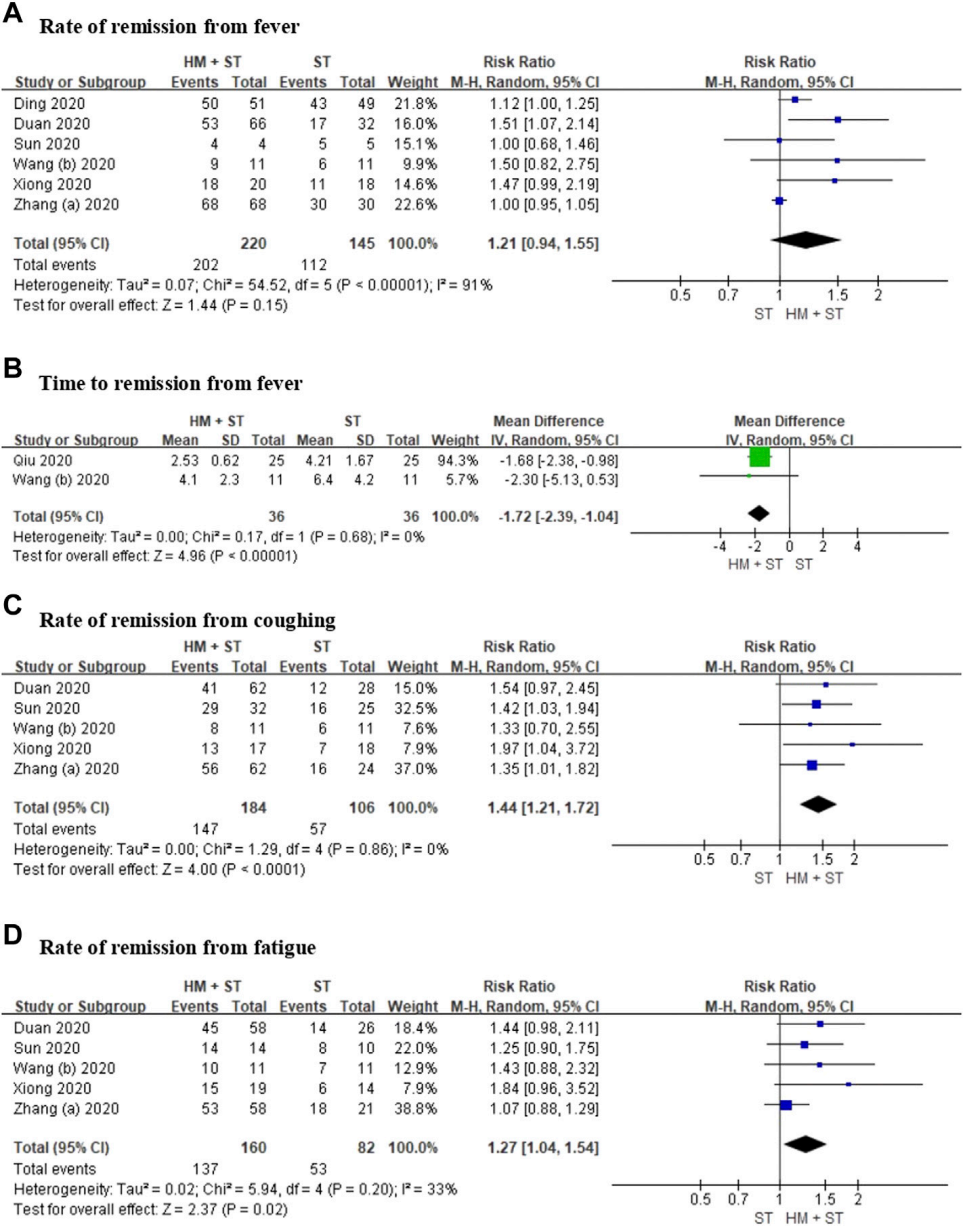
Forest plot for **(A)** rate of remission from fever, **(B)** time to remission from fever, **(C)** rate of remission from coughing, **(D)** rate of remission from fatigue.

Six RCTs including 365 participants, reported the rate of remission from fever. Meta-analysis showed no evidence of a difference between herbal medicine added to standard treatment versus standard treatment for the reduction of fever (RR 1.21, 95% CI 0.94 to 1.55, *p* = 0.15, I^2^ = 91%; low certainty, Figure 3A). In two RCTs including 72 participants, time to remission from fever, using meta-analysis, showed that herbal medicine added to standard treatment reduced time of remission from fever faster compared with standard treatment alone (MD −1.72, 95% CI −2.39 to −1.04, *p* < 0.00001, I^2^ = 0%; low certainty, Figure 3B).

Five RCTs including 290 participants, reported the rate of remission from coughing. Meta-analysis showed that herbal medicine added to standard treatment has a significantly greater effect in alleviating coughing compared with standard treatment (RR 1.44, 95% CI 1.21 to 1.72, *p* < 0.0001, I^2^ = 0%; low certainty, Figure 3C).

Five RCTs including 242 participants, reported rates of remission from fatigue. Meta-analysis showed that herbal medicine added to standard treatment has a significantly greater effect in reducing fatigue compared with standard treatment (RR 1.27, 95% CI 1.04 to 1.54, *p* = 0.02, I^2^ = 33%; low certainty, Figure 3D).

Meta-analysis of two RCTs including 80 participants, also showed that herbal medicine added to standard treatment has a positive effect in reducing sputum production compared with standard treatment (RR 1.73, 95% CI 1.19 to 2.50, *p* = 0.004, I^2^ = 0%; low certainty). In contrast, meta-analyses showed no evidence of a significant difference between herbal medicine added to standard treatment versus standard treatment at reducing symptoms such as a sore throat (three trials, *n* = 31, RR 1.06, 95% CI 0.73 to 1.54, *p* = 0.76, I^2^ = 0%; low certainty), nasal congestion and discharge (two trials, *n* = 14, 95% CI 0.62 to 2.18, *p* = 0.67, I^2^ = 0%; low certainty), diarrhea (two trials, *n* = 28, RR 0.32, 95% CI 0.01 to 8.68, *p* = 0.0.50, I^2^ = 82%; very low certainty), a dry throat (two trials, *n* = 25, RR 0.98, 95% CI 0.65 to 1.48, *p* = 0.93, I^2^ = 0%; very low certainty), and chills (two trials, *n* = 60, RR 0.99, 95% CI 0.75 to 1.32, *p* = 0.97, I^2^ = 0%; low certainty). Forest plots figures are provided in Supplementary Figures 2A–F.

#### Chest Radiological Findings

Eleven RCTs including 1,117 participants, reported the rate of improvement of chest computed tomography (CT) scans. Meta-analysis showed that herbal medicine added to standard treatment was more favorable compared with standard treatment on improving pulmonary lesions (RR 1.23, 95% CI 1.14 to 1.32, *p* < 0.00001, I^2^ = 0%; low certainty, Supplementary Figure S3).

#### Progression to Severe or Critical COVID-19

Seven RCTs including 953 participants, reported the incidence of progression to severe COVID-19 following treatment. Meta-analysis showed that herbal medicine added to standard treatment probably reduced deterioration compared with standard treatment alone (RR 0.52, 95% CI 0.34 to 0.80, *p* = 0.003, I^2^ = 0%; low certainty, Supplementary Figure S4).

#### All-Cause Mortality

Four RCTs including 495 participants, reported mortality. Meta-analysis showed that herbal medicine added to standard treatment probably reduced deaths compared with standard treatment (OR 0.26, 95% CI 0.12 to 0.54, *p* = 0.003, I^2^ = 0%; very low certainty, Supplementary Figure S5).

#### Conversion to a Negative COVID-19 Test

Two RCTs including 162 participants, reported conversion to a negative COVID-19 test, and meta-analyses showed that herbal medicine added to standard treatment probably reduced the conversion time to a negative COVID-19 test compared with standard treatment (MD −3.14, 95% CI −4.68 to −1.60, *p* < 0.0001, I^2^ = 0%; moderate certainty, Supplementary Figure S6A). Meta-analysis of two RCTs including 404 participants, did not show a significant difference for the conversion rate to a negative COVID-19 test between herbal medicine added to standard treatment, versus standard treatment (RR 1.04, 95% CI 0.95 to 1.13, *p* = 0.39, I^2^ = 0%; moderate certainty, Supplementary Figure S6B).

#### Duration of Hospital Stay

Three RCTs including 161 participants, reported the duration of hospital stay. Meta-analysis showed that herbal medicine added to standard treatment reduced the length of stay compared with standard treatment (RR −3.78, 95% CI −5.85 to −1.71, *p* = 0.0003, I^2^ = 0%; low certainty, Supplementary Figure S7).

#### Adverse Events

Seventeen RCTs including 1,716 participants, reported adverse effects relating to herbal medicine and standard treatment intervention. Meta-analysis showed no evidence of a significant difference between herbal medicine added to standard treatment versus standard treatment in the adverse events recorded (RD 0.00, 95% CI -0.04 to 0.03, *p* = 0.88, I^2^ = 75%; low certainty, Supplementary Figure S8). Of the 12 RCTs assessed for adverse events, no severe adverse events were determined in either group. The extracted data for all studies evaluating adverse events are available in Supplementary Table S5.

### Prophylactic Effect of Herbal Medicine

There were no studies that directly addressed the prophylactic potential of herbal medicine intervention in COVID-19.

### Ongoing RCTs of Herbal Medicine Intervention

The ongoing RCTs evaluating herbal medicine for the treatment and prevention of COVID-19 are provided in Supplementary Table S6. As of 28 March 2022, 48 RCTs for the treatment of COVID-19 were identified and one RCT investigating prophylaxis. These RCTs are being conducted or scheduled to start in several countries including China, United States, Singapore, and Pakistan, with expected trial completion dates by December 2022.

## Discussion

### Summary of Main Results

This LSR and meta-analyses presents a comprehensive overview of herbal medicine trials relating to the treatment of COVID-19 up to 28 March 2022. This is the first version of this systematic review where there were 25 published RCTs, and 50 ongoing trials using many different herbal medicine interventions for the treatment and prevention of COVID-19. No studies investigating the effects of herbal medicine in preventing COVID-19 were identified. Of the 25 randomized trials included in this analysis, from a total of 2,288 participants, 1,185 participants were randomized to receive herbal medicine in addition to their standard treatment. The risk of bias of the included RCTs was overall unclear.

Eighteen meta-analyses were performed in this study. Random effects meta-analyses showed evidence of favorable effects of herbal medicine added to standard treatment, versus standard treatment alone based on the total effective rate (*p* = 0.0001), time to remission from fever (*p* < 0.00001), rate of remission from coughing (*p* < 0.0001), fatigue (*p* = 0.02), sputum production (*p* = 0.004), improvement in chest CT scans (*p* < 0.00001), incidence of progression to severe COVID-19 (*p* = 0.003), all-cause mortality (*p* = 0.003), time to a negative COVID-19 test (*p* < 0.0001), and duration of hospital stay (*p* = 0.0003). It was also determined that there was no evidence of a difference between herbal medicine added to standard treatment versus standard treatment alone on the rate of remission from symptoms such as fever, sore throat, nasal congestion and discharge, diarrhea, dry throat, and chills, as well as rate of conversion to a negative COVID-19 test. No severe adverse events were determined relating to herbal medicine interventions, and meta-analysis showed no evidence of a significant difference in the risk of adverse events between the two groups. The findings of this review showed that herbal medicine added to standard treatment may be beneficial for COVID-19 patients, but the certainty of evidence was mostly low to very low.

### Overall Completeness and Applicability of Evidence

Most RCTs included in this study involved participants who were mildly to severely ill with COVID-19. However, different scales of disease severity and progression were used across the studies despite all included studies having originated from China. The severity of COVID-19 of the study participants varied between studies, and the available data was insufficient to allow an evaluation of whether the benefits of herbal medicine would differ with the severity of disease. The outcome measures of the included trials were also complex and varied widely. We selected and analyzed eight outcomes of interest based on “patient-important outcomes” and the general applicability of these outcomes to participants with mild-to-moderate or severe illness. Due to the lack of relevant data in the majority of studies, we could only include limited data for the analysis of safety outcomes. Several RCTs reporting on the outcomes selected for this current review were also excluded in the meta-analyses due to improper data presentation. Additionally, the comparison of differential treatment effects could not be conducted for the types and forms of herbal medicine due to the small number of included studies for each type of herbal medicine.

### Quality of Evidence

The evidence for outcome measures was of moderate to very low certainty, with a vast majority being low to very low certainty. We downgraded the certainty of evidence for most outcomes to low or very low certainty due to the serious risk of bias for inadequate blinding, due to wide CI, or serious inconsistency in statistical heterogeneity (Supplementary Table S4). In addition, the sample size of several studies was small which may have led to higher variability and skewing of findings. Hence, the overall robustness as determined by these review findings was considered to be low.

### Potential Biases in the Review Process

The primary limitation of this review was the unclear risk of bias across the included RCTs, indicating that most studies had methodological limitations. Only one study was judged to have a low risk of bias. All trials were described as “randomized controlled trials” but the reporting of most trials did not comply with the Consolidated Standards of Reporting Trials (CONSORT). Most trials did not provide the relevant information regarding allocation concealment and blinding and, several trials were open-labeled which may introduce bias to the trial results. The risk of bias across the included studies was concerning and resulted in the downgrading of the certainty of evidence. Another limitation was the reliability of the included studies which was possibly reduced due to the lack of trial registration and publicly available study protocol. Where data were missing, we tried to contact all study authors for the detailed reporting of incomplete data but we have yet to receive responses. In this review a comprehensive search was conducted to identify all completed, and ongoing trials, therefore we are confident that most of the relevant studies have been identified in this review, and further monitoring of the list of identified ongoing studies will be performed. We are also aware that excluding preprints from this review may somehow affect the outcome effect size. The evidence in preprints, however, is unstable and often undergoes major changes during peer-reviewed publication. Nevertheless, we will continue to monitor the updates in the evidence reported in preprints and their peer-reviewed publication in order to update the review accordingly.

### Agreement and Disagreements With Other Studies or Reviews

To date, 21 systematic reviews have been identified that have similar findings to this current review (Liu M. et al., 2020; Ang et al., 2020; Sun CY. et al., 2020; Xiong X. et al., 2020; Fan et al., 2020; Pang et al., 2020; Zeng et al., 2020; Zhou et al., 2020; Wang H. et al., 2021; Liu M. et al., 2021; Wang Q. et al., 2021; Du et al., 2021; Fan et al., 2021; Jiang et al., 2021; Li et al., 2021; Liang et al., 2021; Luo et al., 2021; Wu et al., 2021; Yan et al., 2021; Yin et al., 2021; Zhuang et al., 2021). The treatment of COVID-19 benefits from the rapid dissemination of evidence, providing data for the synthesizing of evidence disseminated was constantly complied by systematic reviewers, resulting in a large number of systematic reviews with overlapping evidence, each article updated with the inclusion of only one or two additional studies at the time of publication. The multiplicity of systematic reviews of similar topics can confuse and mislead research end-users. Therefore, the intention in performing a LSR is to provide guidance in the form of a systematic up-to-date review. A comprehensive search (e.g., extensive database search) was performed in this review with analytical methods best suited for the evidence compiled (e.g., random-effects model instead of fixed effect model). This LSR and meta-analyses will be periodically updated with changes from each version highlighted for readers.

### Implication for Practice and Research

Evidence that herbal medicine added to standard treatment of patients with COVID-19 results in important benefits, but the harms to relevant patient-important outcomes are highly uncertain, and not definitive. The evidence regarding the adverse effects of using herbal medicine interventions is also very uncertain due to inconsistent reporting of safety outcomes. Herbal medicine interventions could be recommended after their efficacy and safety concerns are adequately addressed. Amongst the included studies, there was also a lack of consistency in the outcome measurements, where the main outcomes reported varied across the studies and included many insubstantial outcomes. Despite the various challenges faced by COVID-19 clinical research, optimal trial designs of substantial sample size, randomization or blinding methods, and outcome measurements with proper reporting is necessary to increase the quality of evidence. Hence, future well-designed RCTs relating to herbal medicine for the treatment of COVID-19 remains highly anticipated.

## Conclusion

Current evidence suggests that herbal medicine added to standard treatment has potential benefits for treating COVID-19 regardless of the uncertainties. Many research gaps in the clinical studies of herbal medicine still exist and trials with more rigorous study designs are highly warranted.

### Deviation From Registered Protocol

A pre-registered protocol of this LSR is available at the International prospective register of systematic reviews database (PROSPERO CRD42020191711). Considering the inconsistency and complexity of outcome measurements reported amongst the included studies, the planned outcomes list was reviewed and the main outcomes were restricted to patient-important outcomes only. We selected the patient-important outcomes based on the COS that have been registered on the Core Outcome Measures in Effectiveness Trials (COMET) database (Qiu R. et al., 2020; Jin et al., 2020; Marshall et al., 2020; Tong et al., 2020). Subsequent to the originally planned risk of bias assessment, we performed an additional assessment on the quality of evidence using the GRADEpro guideline development tool. These changes were implemented before the data analysis.

## Data Availability

The original contributions presented in the study are included in the article/Supplementary Material, further inquiries can be directed to the corresponding author.
