# Supplementary material for: Herbal Medicine Intervention for the Treatment of COVID-19: A Living Systematic Review and Cumulative Meta-Analysis
Source: Front Pharmacol. 2022 Jun 20;13:906764. doi: 10.3389/fphar.2022.906764 (PMC9251500; doi:10.3389/fphar.2022.906764)
Supplement: Supplementary file 1 [file DataSheet1.docx]

Supplementary Material

**Supplementary Figure 1. Risk of bias of included studies**

1. **
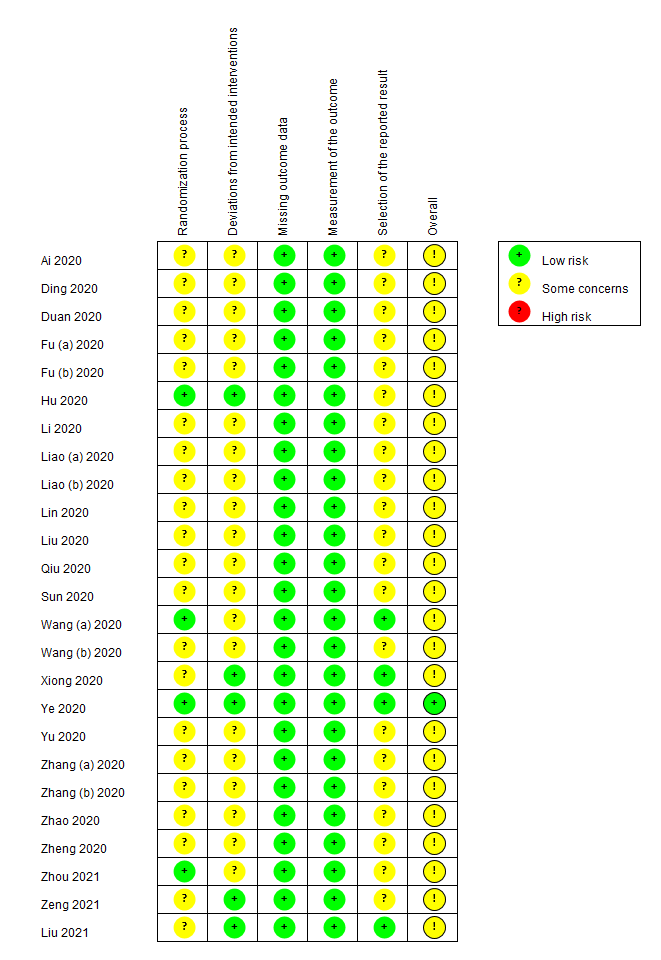
Risk of bias graph (B) Risk of bias summary**

**
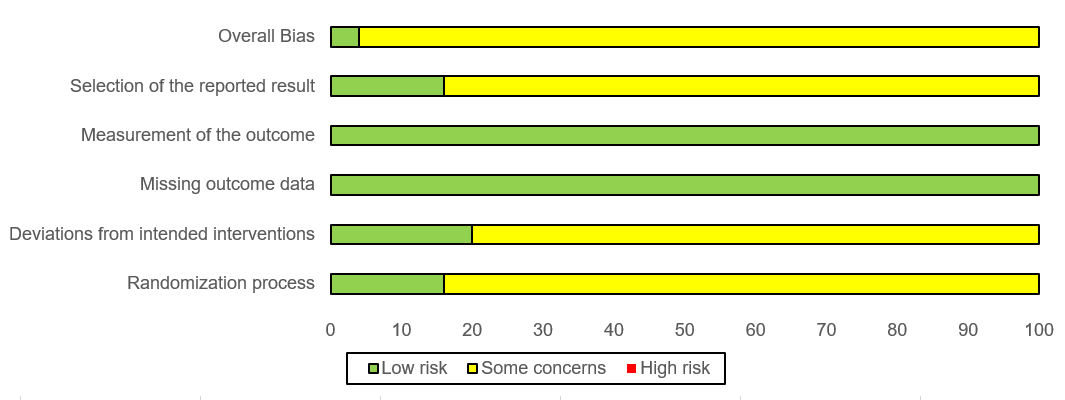
**

**Supplementary Figure 2A−F. Forest plots for COVID-19 symptom resolution**

1. **Sputum production**

**
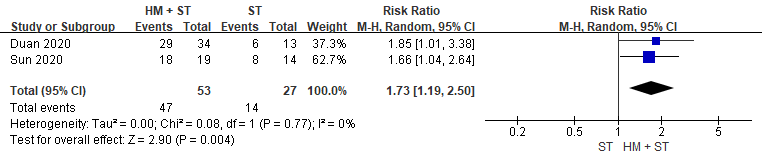
**

1. **Sore throat**


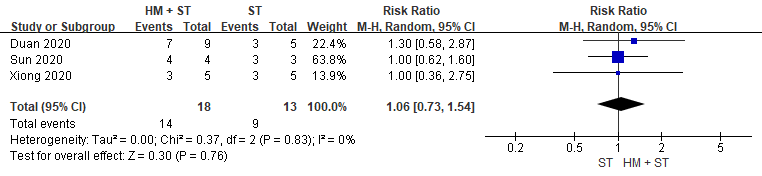


1. **Nasal congestion and runny nose**

**
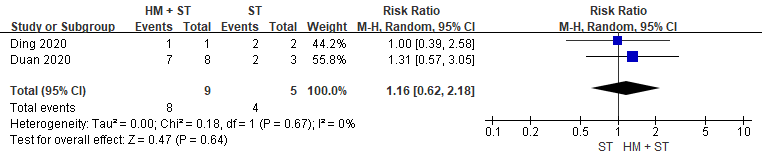
**

1. **Diarrhea**


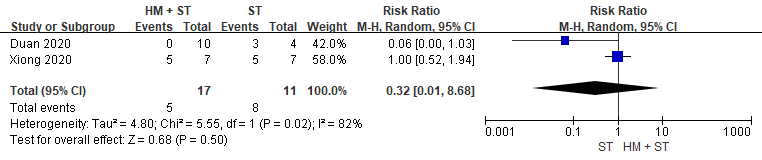


1. **Dry throat**


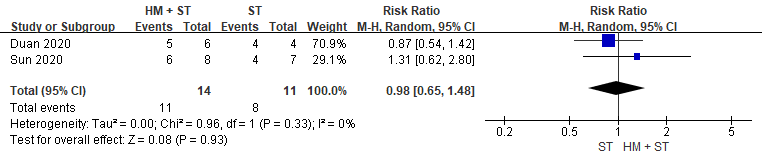


1. **Chills**

**
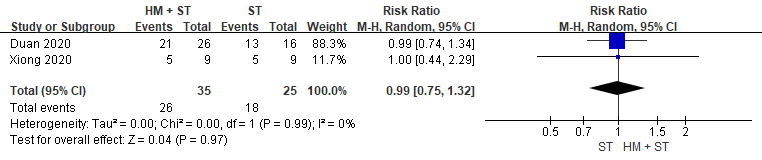
**

**Supplementary Figure 3. Forest plot for chest radiological findings due to COVID-19**

**
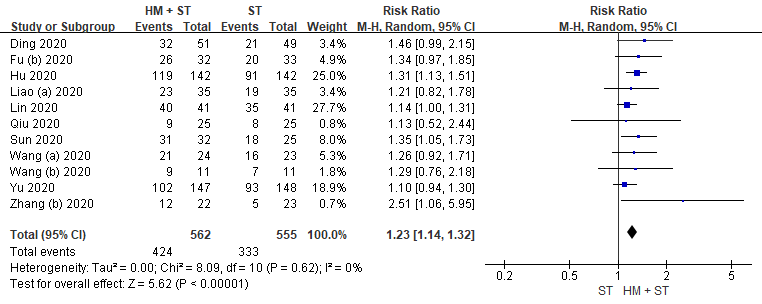
**

**Supplementary Figure 4. Forest plot for progression to severe or critical COVID-19**

**
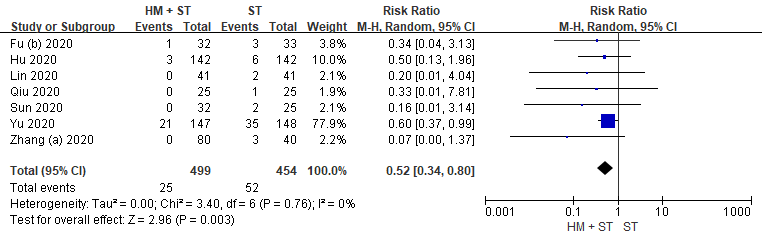
**

**Supplementary Figure 5. Forest plot for all-cause mortality from COVID-19**

**
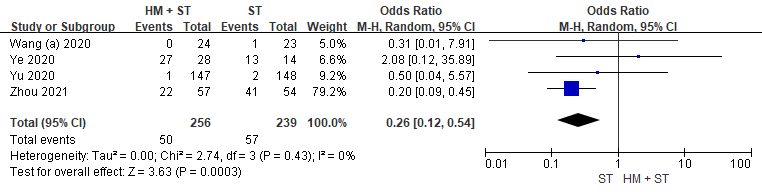
**

**Supplementary Figure 6. Forest plots for negative COVID-19 tests**

1. **Time to conversion to negative COVID-19 test**

**
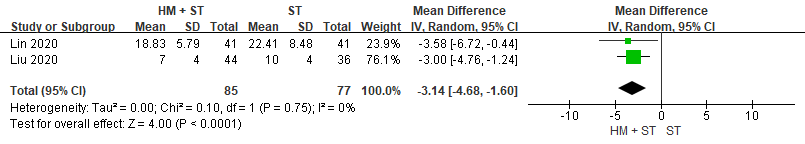
**

1. **Rate of conversion to negative COVID-19 test**

**
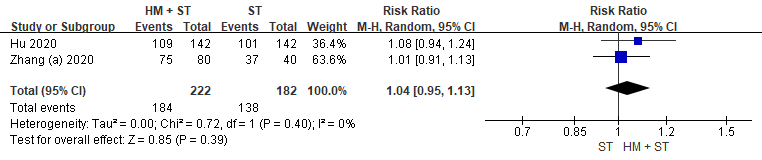
**

**Supplementary Figure 7. Forest plot for duration of hospital stay due to COVID-19**

**
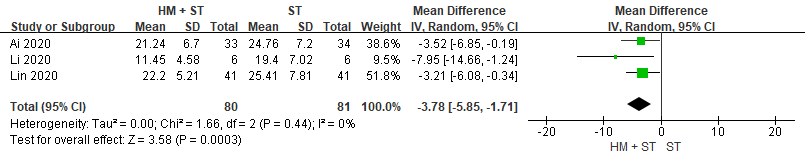
**

**Supplementary Figure 8. Forest plot for adverse events from treatment of COVID-19**

**
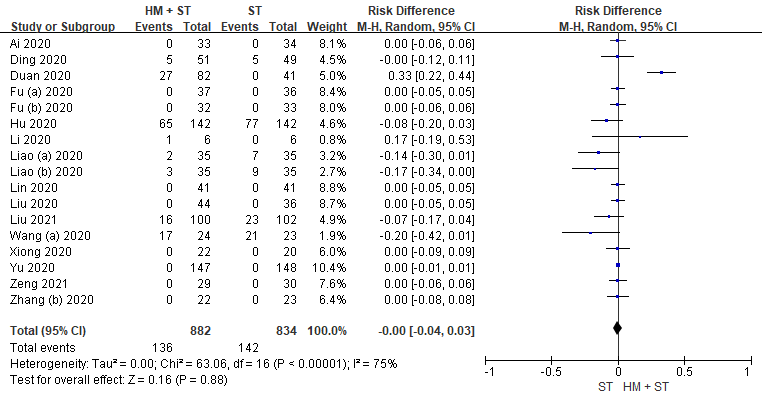
**

**Supplementary Table 1. Search strategy**

| English | 1 (“herbal medicine” OR “traditional medicine” OR “oriental medicine” OR “Chinese medicine” OR “Korean medicine” OR “herbal formula” OR herb* OR Chinese Patent Medicine* OR Alternative Medicine* OR Alternative Therap* OR Complementary Medicine* or Complementary Therap* OR Integrat* Medicine* OR Integrat* Therap* OR (Traditional adj3 (Medicine or Therap*)) OR (Traditional Chinese adj3 Medicine*))  2 (“coronavirus disease 2019” OR “COVID-2019” OR “2019 novel coronavirus” OR “2019-nCoV” OR “Novel Coronavirus Pneumonia” OR “NCP” OR “Severe acute respiratory syndrome coronavirus 2” OR “SARS CoV-2” OR “new coronavirus” OR “novel coronavirus”)  3 1 and 2  4 (Clinical Trials, Randomized) or (Controlled Clinical Trials, Randomized) or randomized controlled trial or RCT or (Trials, Randomized Clinical)  5 3 and 4 |
| --- | --- |
| Chinese | 1 新型冠状病毒肺炎 OR 新型冠状病毒  2 中药 OR 草药 OR 中医 OR 传统医疗 OR 替代疗法 OR 中西医结合 OR 补充疗法  3 临床 OR 临床研究 OR 临床疗效 OR 随机 OR 对照  4 1 and 2 and 3 |

**Supplementary Table 2. Reasons for exclusion of studies from this review**

| Study ID | Title of excluded studies | Reasons |
| --- | --- | --- |
| Wang 2020 | Clinical study of Gegen Qinlian pill in treating COVID-19 | Both intervention and control group were intervened with combination of herbal medicine and standard medicine |
| Shi 2021 | Clinical studies of comprehensive TCM treatment to 30 cases of Qi Yin deficiency type of COVID-19 in its recovery period | Trial participants were in recovery stage |
| Li 2021 | A preliminary study of traditional Chinese medicine promoting health recovery after COVID-19 | Trial participants were in recovery stage |
| Wang 2020 | Clinical effect of the treatment of novel coronavirus pneumonia by internal administration of traditional Chinese medicine plus fumigation and absorption combined with super dose of vitamin C in treating COVID-19 | Two intervention arms were intervened with two other types of TCM intervention (pre-defined exclusion criteria) |
| Wang 2020 | Lianhua Qingwen capsule and interferon-α combined with lopinavir/ritonavir for the treatment of 30 COVID-19 patients | Both intervention and control group were intervened with same type of herbal medicine but different type of standard medicine |
| Meng 2020 | The effect of comprehensive traditional Chinese medicine nursing on improving symptoms and quality of life of patients with new coronary pneumonia | Trial focused on patient care |
| Lv 2020 | A clinical observation of 63 cases of novel coronavirus pneumonia suspected cases treated with Lianhua Qingwen granule | Only suspected cases were included as trial participants |
| Xiao 2020 | Efficacy of Huoxiang Zhengqi dropping pills and Lianhua Qingwen granules in treatment of COVID-19: A randomized controlled trial | Both suspected cases and diagnosed cases were included as trial participants, and the data provided could not be separated |
| Ai 2020 | Effect of integrated traditional Chinese and Western medicine on T lymphocyte subsets of patients with normal type of COVID-19 | Different treatment regimens were performed at different stages of the trial with different exposure length |
| Tian 2020 | Clinical study on the treatment of novel Coronavirus pneumonia using integrated traditional Chinese and Western medicine | Both intervention and control group were intervened with combination of herbal medicine and standard medicine |
| Zhou 2020 | Clinical value of Diammonium Glycyrrhizinate in treatment of COVID-19 | Herbal extractions (pre-defined exclusion criteria) |
| Mesri 2021 | The effects of combination of Zingiber officinale and Echinacea on alleviation of clinical symptoms and hospitalization rate of suspected COVID-19 outpatients: a randomized controlled trial | Herbal extractions (pre-defined exclusion criteria) |
| Pawar 2021 | Oral Curcumin with Piperine as Adjuvant Therapy for the Treatment of COVID-19: A Randomized Clinical Trial | Herbal extractions (pre-defined exclusion criteria) |
| Zhang 2021 | Efficacy and safety of Xiyanping injection in the treatment of COVID-19: A multicenter, prospective, open-label and randomized controlled trial | Herbal injections (pre-defined exclusion criteria) |
| Wen 2020 | Effect of Xuebijing injection on inflammatory markers and disease outcome of coronavirus disease 2019 | Herbal injections (pre-defined exclusion criteria) |
| Xu 2021 | Efficacy and safety of Reduning injection in the treatment of COVID-19: a randomized, multicenter clinical study | Herbal injections (pre-defined exclusion criteria) |
| Ni 2021 | Effects of Shuanghuanglian oral liquids on patients with COVID-19: a randomized, open-label, parallel-controlled, multicenter clinical trial | Multiple arms with different dosages of herbal medicine |
| Yan 2020 | Large-scale prospective clinical study on prophylactic intervention of COVID-19 in community population using Huoxiang Zhengqi Oral Liquid and Jinhao Jiere Granules | Inappropriate outcomes |
| Liu 2019 | Efficacy of No. 1 pneumonia prescription in the treatment of Corona Virus Disease | Article retracted by author |
| Koshak 2021 | Nigella sativa for the treatment of COVID-19: An open-label randomized controlled clinical trial | Herbal extractions (pre-defined exclusion criteria) |
| Yan 2021 | Effects and safety of herbal medicines among community-dwelling residents during COVID-19 pandemic: A large prospective, randomized controlled trial (RCT) | Inappropriate outcomes |
| Chen 2022 | Efficacy and safety of Bufei Huoxue capsules in the management of convalescent patients with COVID-19 infection: A multicentre, double-blind, and randomised controlled trial. | Trial participants were in recovery stage. |
| Li 2021 | Safety and efficacy of artemisinin-piperaquine for treatment of COVID-19: an open-label, non-randomised and controlled trial | Herbal extractions (pre-defined exclusion criteria) |
| Devpura 2021 | Randomized placebo-controlled pilot clinical trial on the efficacy of ayurvedic treatment regime on COVID-19 positive patients | Both intervention and control group were intervened with herbal medicine |
| Natarajan 2021 | Kabasura Kudineer (KSK), a poly-herbal Siddha medicine, reduced SARS-CoV-2 viral load in asymptomatic COVID-19 individuals as compared to vitamin C and zinc supplementation: findings from a prospective, exploratory, open-labeled, comparative, randomized controlled trial, Tamil Nadu, India | Inappropriate comparison group |
| Karimi 2021 | Efficacy of Persian medicine herbal formulations (capsules and decoction) compared to standard care in patients with COVID-19, a multicenter open-labeled, randomized, controlled clinical trial | Intervention group were intervened with combination of several types of herbal medicine |

**Supplementary Table 3. Risk of bias for the included studies in the review**

|  | 1.1 | 1.2 | 1.3 | R1 | 2.1. | 2.2 | 2.3 | 2.4 | R2 | 3.1 | R3 | 4.1 | 4.2 | 4.3 | 4.4 | 4.5 | R4 | 5.1 | 5.2 | 5.3 | R5 | Overall ROB |
| --- | --- | --- | --- | --- | --- | --- | --- | --- | --- | --- | --- | --- | --- | --- | --- | --- | --- | --- | --- | --- | --- | --- |
| Ai 2020 | Y (Random number table) | NI | N | SC | NI | NI | NI | Y (ITT analysis) | SC | Y (All) | L | N | N | NI | N | N | L | NI (Trial protocol is not available) | N | N | SC | SC |
| Ding 2020 | Y (Computer-generated random numbers) | NI | N | SC | NI | NI | NI | Y (ITT analysis) | SC | Y (All) | L | N | N | NI | N | N | L | NI (Trial protocol is not available) | N | N | SC | SC |
| Duan 2020 | Y (Computer-generated random numbers) | NI | N | SC | NI | Y | NI | Y (ITT analysis) | SC | Y (All) | L | N | N | N (Outcomes were assessed by other physician) |  | | L | NI (Trial protocol is not available) | N | N | SC | SC |
| Fu (a) 2020 | PY (Absence of specific information) | NI | N | SC | NI | NI | NI | Y (ITT analysis) | SC | Y (All) | L | N | N | NI | N | N | L | NI (Trial protocol is not available) | N | N | SC | SC |
| Fu (b) 2020 | Y (Random number table) | NI | N | SC | NI | NI | NI | Y (ITT analysis) | SC | Y (All) | L | N | N | NI | N | N | L | NI (Trial protocol is not available) | N | N | SC | SC |
| Hu 2020 | Y (Computer-generated random numbers) | Y (allocation controlled by independent unit) | N | L | N (Open-labelled) | N | NI | Y (Both ITT and PP analyses) | L | Y (Loss to follow-up is less than 20%) | L (Missing outcome data are unlikely to be related to true outcome) | N | N | Y (Open-labelled) | N | N | L | N (Routine blood test and biochemical indicators were listed as secondary outcome in trial protocol but were eluded from reported outcome) | N | N | SC | SC |
| Li 2020 | PY (Absence of specific information) | NI | N | SC | NI | NI | NI | Y (ITT analysis) | SC | Y (All) | L | N | N | NI | N | N | L | NI (Trial protocol is not available) | N | N | SC | SC |
| Liao (a) 2020 | PY (Absence of specific information) | NI | N | SC | NI | NI | NI | Y (ITT analysis) | SC | Y (All) | L | N | N | NI | N | N | L | NI (Trial protocol is not available) | N | N | SC | SC |
| Liao (b) 2020 | PY (Absence of specific information) | NI | N | SC | NI | NI | NI | Y (ITT analysis) | SC | Y (All) | L | N | N | NI | N | N | L | NI (Trial protocol is not available) | N | N | SC | SC |
| Lin 2020 | Y (Random number table) | NI | N | SC | NI | NI | NI | Y (ITT analysis) | SC | Y (All) | L | N | N | NI | N | N | L | NI (Trial protocol is not available) | N | N | SC | SC |
| Liu 2020 | PY (Absence of specific information) | NI | N | SC | NI | NI | NI | Y (ITT analysis) | SC | Y (All) | L | N | N | NI | N | N | L | NI (Trial protocol is not available) | N | N | SC | SC |
| Qiu 2020 | Y (Random number table) | NI | N | SC | NI | NI | NI | Y (ITT analysis) | SC | Y (All) | L | N | N | NI | N | N | L | NI (Trial protocol is not available) | N | N | SC | SC |
| Sun 2020 | Y (Random number table) | NI | N | SC | N (Open-labelled) | N | NI | Y (ITT analysis) | SC | Y (All) | L | N | N | NI | N | N | L | NI (Trial protocol is not available) | N | N | SC | SC |
| Wang (a) 2020 | Y (Computer-generated random numbers) | Y (allocation controlled by independent unit) | N | L | Y (Double-blinded) | PY |  | PN (PP analysis) | SC | Y (Loss to follow-up is less than 20%) | L (Missing outcome data are unlikely to be related to true outcome) | N | N | N (The data were evaluated by a specialist in a blinded fashion) |  | | L | Y (The outcome measures and analyses reported were consistent with the trial protocol) | N | N | L | SC |
| Wang (b) 2020 | Y (Random number table) | NI | N | SC | N (Open-labelled) | N | NI | Y (ITT analysis) | SC | Y (All) | L | N | N | Y (Open-labelled) | N | N | L | NI (Trial protocol is not available) | N | N | SC | SC |
| Xiong 2020 | Y (Coin tossing) | NI | N | SC | NI | NI | NI | Y (ITT analysis) | L | Y (All) | L | N | N | NI | N | N | L | Y (The outcome measures and analyses reported were consistent with the trial protocol) | N | N | L | SC |
| Ye 2020 | Y (Simple random allocation method) | Y (Concealed to laboratory personnel and outcome assessors) | N | L | N (Open-labelled) | Y | NI | Y (ITT analysis) | L | Y (Loss to follow-up is less than 20%) | L (Missing outcome data are unlikely to be related to true outcome) | N | N | N (Allocation was concealed to outcome assessors) |  | | L | Y (The outcome measures and analyses reported were consistent with the trial protocol) | N | N | L | L |
| Yu 2020 | Y (Random number table) | NI | N | SC | NI | NI | NI | Y (ITT analysis) | SC | Y (All) | L | N | N | NI | N | N | L | NI (Trial protocol is not available) | N | N | SC | SC |
| Zhang (a) 2020 | PY (Absence of specific information) | NI | N | SC | NI | NI | NI | Y (ITT analysis) | SC | Y (All) | L | N | N | NI | N | N | L | NI (Trial protocol is not available) | N | N | SC | SC |
| Zhang (b) 2020 | PY (Absence of specific information) | NI | N | SC | NI | NI | NI | Y (ITT analysis) | SC | Y (All) | L | N | N | NI | N | N | L | NI (Trial protocol is not available) | N | N | SC | SC |
| Zhao 2020 | PY (Absence of specific information) | NI | N | SC | NI | NI | NI | Y (ITT analysis) | SC | Y (All) | L | N | N | NI | N | N | L | N (Trial protocol is not available and selective reporting for intended outcome measurements) | N | N | SC | SC |
| Zheng 2020 | PY (Absence of specific information) | NI | N | SC | NI | NI | NI | Y (ITT analysis) | SC | Y (All) | L | N | N | NI | N | N | L | NI (Trial protocol is not available) | N | N | SC | SC |
| Zhou 2021 | Y (Computer-generated random numbers) | Y (Individually numbered packs) | N | L | NI | NI | NI | PN (PP analysis) | SC | Y (Loss to follow-up is less than 20%) | L (Missing outcome data are unlikely to be related to true outcome) | N | N | NI | N | N | L | N (Reported results is less detailed compared to intended outcome measurements) | N | N | SC | SC |
| Zeng 2021 | Y (Computer-generated random numbers) | NI | N | SC | N (Open-labelled) | Y | NI | Y (ITT analysis) | L | Y (Loss to follow-up is less than 20%) | L (Missing outcome data are unlikely to be related to true outcome) | N | N | NI | N | N | L | N (Reported results is slightly different compared to intended outcome measurements) | N | N | SC | SC |
| Liu 2021 | Y (Computer-generated random numbers) | NI | N |  | N (Open-labelled) | Y | NI | Y (Both ITT and PP analysis) | L | Y (Loss to follow-up is less than 20%) | L (Missing outcome data are unlikely to be related to true outcome) | N | N | NI | N | N | L | (The outcome measures and analyses reported were consistent with | N | N | L | SC |

1.1: Was the allocation sequence random?

1.2: Was the allocation sequence concealed until participants were enrolled and assigned to interventions?

1.3: Did baseline differences between intervention groups suggest a problem with the randomization process?

R1: Algorithm result for ‘Randomization process’

2.1: Were participants aware of their assigned intervention during the trial?

2.2: Were carers and people delivering the interventions aware of participants' assigned intervention during the trial?

2.3: Were there deviations from the intended intervention that arose because of the experimental context?

2.4: Was an appropriate analysis used to estimate the effect of assignment to intervention? R2: Algorithm result for ‘Deviations from intended interventions’

3.1: Were data for this outcome available for all, or nearly all, participants randomized?

R3: Algorithm result for ‘Missing outcome data’

4.1: Was the method of measuring the outcome inappropriate?

4.2: Could measurement or ascertainment of the outcome have differed between intervention groups?

4.3: Were outcome assessors aware of the intervention received by study participants?

4.4: Could assessment of the outcome have been influenced by knowledge of intervention received?

4.5: Is it likely that assessment of the outcome was influenced by knowledge of intervention received?

R4: Algorithm result for ‘Measurement of the outcome’

5.1: Were the data that produced this result analysed in accordance with a pre-specified analysis plan that was finalized before unblinded outcome data were available for analysis?

5.2: ... multiple eligible outcome measurements (e.g., scales, definitions, time points) within the outcome domain?

5.3: ... multiple eligible analyses of the data?

R5: Algorithm result for ‘Selection of the reported result’

**Supplementary Table 4. Summary of findings to determine the certainty of evidence (GRADE)**

| Outcomes | № of participants  (studies) Follow up | Certainty of the evidence (GRADE) | Relative effect (95% CI) | Anticipated absolute effects | |
| --- | --- | --- | --- | --- | --- |
|  |  |  |  | Risk with standard care alone | Risk difference with herbal medicine added on standard care |
| Total effective rate | 1192 (10 RCTs) | ⊕⊕◯◯ LOW ^a,b,c^ | RR 1.21 (1.10 to 1.33) | 680 per 1,000 | 143 more per 1,000 (68 more to 225 more) |
| Rate of symptom resolution for fever | 365 (6 RCTs) | ⊕⊕◯◯ LOW ^a,b,c^ | RR 1.21 (0.94 to 1.55) | 772 per 1,000 | 162 more per 1,000 (46 fewer to 425 more) |
| Time to symptom resolution for fever | 72 (2 RCTs) | ⊕⊕◯◯ LOW ^a,d^ | - | The mean time to complete recovery for fever was 0 | MD 1.72 lower (2.39 lower to 1.04 lower) |
| Rate of symptom resolution for cough | 290 (5 RCTs) | ⊕⊕◯◯ LOW ^a,d^ | RR 1.44 (1.21 to 1.72) | 538 per 1,000 | 237 more per 1,000 (113 more to 387 more) |
| Rate of symptom resolution for fatigue | 242 (5 RCTs) | ⊕⊕◯◯ LOW ^a,d^ | RR 1.27 (1.04 to 1.54) | 646 per 1,000 | 175 more per 1,000 (26 more to 349 more) |
| Rate of symptom resolution for sore throat | 31 (3 RCTs) | ⊕⊕◯◯ LOW ^a,e^ | RR 1.06 (0.73 to 1.54) | 692 per 1,000 | 42 more per 1,000 (187 fewer to 374 more) |
| Rate of symptom resolution for nasal congestion and runny nose | 14 (2 RCTs) | ⊕⊕◯◯ LOW ^a,e^ | RR 1.16 (0.62 to 2.18) | 800 per 1,000 | 128 more per 1,000 (304 fewer to 944 more) |
| Rate of symptom resolution for diarrhea | 28 (2 RCTs) | ⊕◯◯◯ VERY LOW ^a,c,d,e^ | RR 0.32 (0.01 to 8.68) | 727 per 1,000 | 495 fewer per 1,000 (720 fewer to 5,585 more) |
| Rate of symptom resolution for dry throat | 25 (2 RCTs) | ⊕◯◯◯ VERY LOW ^a,b,e^ | RR 0.98 (0.65 to 1.48) | 727 per 1,000 | 15 fewer per 1,000 (255 fewer to 349 more) |
| Rate of symptom resolution for chills | 60 (2 RCTs) | ⊕⊕◯◯ LOW ^a,d^ | RR 0.99 (0.75 to 1.32) | 720 per 1,000 | 7 fewer per 1,000 (180 fewer to 230 more) |
| Rate of symptom resolution for sputum production | 80 (2 RCTs) | ⊕⊕◯◯ LOW ^a,d^ | RR 1.73 (1.19 to 2.50) | 519 per 1,000 | 379 more per 1,000 (99 more to 778 more) |
| Chest radiological findings | 1117 (11 RCTs) | ⊕⊕◯◯ LOW ^a,b^ | RR 1.23 (1.14 to 1.32) | 600 per 1,000 | 138 more per 1,000 (84 more to 192 more) |
| Progression to severe or critical COVID-19 | 953 (7 RCTs) | ⊕⊕◯◯ LOW ^a,d^ | RR 0.52 (0.34 to 0.80) | 115 per 1,000 | 55 fewer per 1,000 (76 fewer to 23 fewer) |
| All-cause mortality | 495 (4 RCTs) | ⊕◯◯◯ VERY LOW ^a,b,d^ | OR 0.26 (0.12 to 0.54) | 238 per 1,000 | 163 fewer per 1,000 (202 fewer to 94 fewer) |
| Rate to a negative COVID-19 coronavirus test | 404 (2 RCTs) | ⊕⊕⊕◯ MODERATE ^a^ | RR 1.04 (0.95 to 1.13) | 758 per 1,000 | 30 more per 1,000 (38 fewer to 99 more) |
| Time to a negative COVID-19 coronavirus test | 162 (2 RCTs) | ⊕⊕⊕◯ MODERATE ^a^ | - | The mean time to a negative COVID-19 coronavirus test was 0 | MD 3.14 lower (4.68 lower to 1.6 lower) |
| Duration of hospital stay | 161 (3 RCTs) | ⊕⊕◯◯ LOW ^a,d^ | - | The mean length of hospital stay was 0 | MD 3.78 lower (5.85 lower to 1.71 lower) |
| Adverse events | 1716 (17 RCTs) | ⊕⊕◯◯ LOW ^a,b,d^ | OR 0.69  (0.28 to 1.70) | 143 per 1,000 | 40 fewer per 1,000 (98 fewer to 78 more) |
| *The risk in the intervention group (and its 95% confidence interval) is based on the assumed risk in the comparison group and the relative effect of the intervention (and its 95% CI).  CI: Confidence interval; RR: Risk ratio; MD: Mean difference; OR: Odds ratio | | | | | |
| GRADE Working Group grades of evidence High certainty: We are very confident that the true effect lies close to that of the estimate of the effect Moderate certainty: We are moderately confident in the effect estimate: The true effect is likely to be close to the estimate of the effect, but there is a possibility that it is substantially different Low certainty: Our confidence in the effect estimate is limited: The true effect may be substantially different from the estimate of the effect Very low certainty: We have very little confidence in the effect estimate: The true effect is likely to be substantially different from the estimate of effect | | | | | |

**Explanations**

a. Unclear risk of bias across studies in general. Most studies are lacking substantial information for allocation concealment and blinding which might lead to judgement of high bias despite of apparently sound randomization method.

b. The point of estimates and the overlap of confidence intervals are inconsistent.

c. High heterogeneity was determined.

d. Confidence intervals (CI) around the estimates of treatment effect are considerably wide.

e. Sample size was small which resulted in meaning benefits.

**Supplementary Table 5. Details on adverse events**

| Study, year | Adverse events |
| --- | --- |
| Ding 2020 | Low blood counts: (A) 3, (B) 2; Renal dysfunction: (A) 2, (B) 3 |
| Li 2020 | Skin itch: (A) 1 |
| Liao (a) 2020 | Skin rash: (A) 1, (B) 2; Insomnia: (B) 1; Tremor: (B) 1; Skin itch: (A) 1; (B) 3 |
| Liao (b) 2020 | Skin rash: (B) 1; Insomnia: (A) 1, (B) 4; Tremor: (A) 1, (B) 2; Skin itch: (A) 1, (B) 2 |
| Lin 2020 | No cases of AEs |
| Xiong 2020 | No cases of AEs |
| Zeng 2021 | No cases of AEs |
| Zhang 2020 (b) | No cases of AEs |
| Ai 2020 | No cases of AEs |
| Duan 2020 | Diarrhea: (A) 27 |
| Fu (a) 2020 | No cases of AEs |
| Fu (b) 2020 | No cases of AEs |
| Liu 2020 | No cases of AEs |
| Yu 2020 | No cases of AEs |
| Zhou 2020 | Side effects instead of adverse events were reported |
| Liu 2021 | Diarrhea: (A) 8, (B) 7; Abdominal discomfort: (A) 2, (B) 3; Decreased appetite: (A) 3; Anxiety: (B) 2; Oral ulcer: (A) 1, (B) 1; Short breath: (B) 2; Constipation: (B) 1; Vomiting: (A) 1; Itchy skin: (B) 1; Lower extremity edema: (B) 1; Dry eye: (A) 1; Limb pain: (B) 1 |
| Hu 2020 | Abnormal liver function: (A) 32, (B) 32; Renal dysfunction: (A) 8, (B) 11; Headache: (A) 1, (B) 1; Nausea: (A) 6, (B) 5; Vomiting: (A) 2, (B) 3; Diarrhea: (A) 8, (B) 19; Loss of appetite: (A) 8, (B) 6 |
| Wang (a) 2020 | Diarrhea: (A) 9, (B) 8; Anorexia: (A) 4, (B) 5; Nausea: (A) 2, (B) 3; Stomach pain: (A) 2, (B) 3; Allergic reaction: (B) 1; Sepsis: (B) 1 |

**Supplementary Table 6. Ongoing RCTs of herbal medicine intervention**

| Identifier | Estimate enrollment (N) | Intervention | Control | Country where the trial was conducted | Status | Estimated primary completion date | Study type |
| --- | --- | --- | --- | --- | --- | --- | --- |
| NCT04939415 | 66 | (A) HM (modified Qingfei Paidu capsules) | (B) Placebo | United states | Recruiting | July 1, 2022 | Treatment |
| NCT04723524 | 300 | (A) HM (Jinhua Qinggan granules) | (B) Placebo | Pakistan | Recruiting | June 2021 | Treatment |
| NCT04291053 | 550 | (A) HM (Huaier granules), plus B | (B) Standard care | China | Not yet recruiting | August 1, 2020 | Treatment |
| NCT04433013 | 300 | (A) HM (Lianhua Qingwen capsules) | (B) Placebo | Singapore | Not yet recruiting | December 2020 | Treatment |
| NCT04967755 | 200 | (A) HM (JingSi herbal tea), plus B | (B) Standard care | Taiwan | Recruiting | August 31, 2021 | Treatment |
| NCT04810689 | 60 | (A) HM (Xuanfei Baidu granules) | (B) Placebo | United states | Recruiting | March 1, 2022 | Treatment |
| ChiCTR2000029434 | 240 | (A) HM (Lianhua Qingwen capsules), plus B | (B) Standard care | China | Completed | April 30, 2020 | Treatment |
| ChiCTR2000029438 | 100 | (A) HM plus B | (B) Standard care | China | Not yet recruiting | December 1, 2021 | Treatment |
| ChiCTR2000029439 | 120 | (A) HM plus B | (B) Standard care | China | Not yet recruiting | December 31, 2021 | Treatment |
| ChiCTR2000029461 | 100 | (A) HM plus B | (B) Standard care | China | Not yet recruiting | December 31, 2021 | Treatment |
| ChiCTR2000029518 | 140 | (A) HM plus B | (B) Standard care | China | Recruiting | April 30, 2020 | Treatment |
| ChiCTR2000029549 | 400 | (A) HM plus B | (B) Standard care | China | Recruiting | May 1, 2020 | Treatment |
| ChiCTR2000029747 | 200 | (A) HM | (B) Standard care | China | Recruiting | February 10, 2021 | Treatment |
| ChiCTR2000029755 | 120 | (A) HM (Jinyebaidu granules), plus B | (B) Standard care | China | Recruiting | May 30, 2020 | Treatment |
| ChiCTR2000029763 | 408 | (A) HM plus B | (B) Standard care | China | Recruiting | May 31, 2020 | Treatment |
| ChiCTR2000029769 | 40 | (A) HM (Babaodan), plus B | (B) Standard care | China | Not yet recruiting | April 30, 2020 | Treatment |
| ChiCTR2000029788 | 60 | (A) HM plus B | (B) Standard care | China | Not yet recruiting | December 30, 2021 | Treatment |
| ChiCTR2000029790 | 120 | (A) HM plus B | (B) Standard care | China | Recruiting | October 31, 2020 | Treatment |
| ChiCTR2000029813 | 72 | (A) HM (Tanreqing capsules), plus B | (B) Standard care | China | Recruiting | August 14, 2020 | Treatment |
| ChiCTR2000029869 | 300 | (A) HM (Baidu Jieduan formula), plus B | (B) Standard care | China | Not yet recruiting | June 30, 2020 | Treatment |
| ChiCTR2000029941 | 200 | (A) HM (Zhongyao Fufang granules), plus B | (B) Standard care | China | Not yet recruiting | June 1, 2020 | Treatment |
| ChiCTR2000029960 | 100 | (A) HM plus B | (B) Standard care | China | Not yet recruiting | May 31, 2020 | Treatment |
| ChiCTR2000030034 | 132 | (A) HM plus B | (B) Standard care | China | Recruiting | June 30, 2020 | Treatment |
| ChiCTR2000030166 | 20 | (A) HM (Qingwen Baidu Yin granules), plus B | (B) Standard care | China | Not yet recruiting | May 14, 2020 | Treatment |
| ChiCTR2000030188 | 120 | (A) HM plus B | (B) Standard care | China | Recruiting | March 30, 2020 | Treatment |
| ChiCTR2000030469 | 90 | (A) HM (Liu Shen Wan), plus B | (B) Standard care | China | Recruiting | May 27, 2020 | Treatment |
| ChiCTR2000030479 | 100 | (A) HM (Yiqi Huashi Jiedu Fang), plus B | (B) Standard care | China | Not yet recruiting | February 25, 2021 | Treatment |
| ChiCTR2000030522 | 100 | (A) HM (Ma Xing Shi Gan Tang), plus B | (B) Placebo | China | Completed | September 9, 2020 | Treatment |
| ChiCTR2000030936 | 284 | (A) HM (Xinguan No. 2/Xinguan No. 3), plus B | (B) Standard care | China | Recruiting | May 10, 2020 | Treatment |
| ChiCTR2000030988 | 204 | (A) HM (Hua Shi Bai Du granules), plus B | (B) Standard care | China | Not yet recruiting | May 31, 2020 | Treatment |
| ChiCTR2000033320 | 300 | (A) HM | (B) Standard care | China | Recruiting | December 31, 2020 | Treatment |
| ChiCTR2000029993 | 40 | (A) HM (Liushen capsules) | (B) Standard care | China | Recruiting | August 20, 2020 | Treatment |
| ChiCTR2000029855 | 180 | (A) HM (Qingfei prescription) | (B) Standard care | China | Recruiting | Not reported | Treatment |
| ChiCTR2000029819 | 80 | (A) HM (Babaodan), plus B | (B) Standard care | China | Recruiting | August 11, 2020 | Treatment |
| ChiCTR2000029814 | 30 | (A) HM plus B | (B) Standard care | China | Recruiting | December 31, 2020 | Treatment |
| ChiCTR2000029778 | 600 | (A) HM (Qingfei Paidu decoction/Shufeng Jiedu capsules), plus B | (B) Standard care | China | Recruiting | May 13, 2020 | Treatment |
| ChiCTR2000029751 | 350 | (A) HM plus B | (B) Standard care | China | Recruiting | December 31, 2020 | Treatment |
| ChiCTR2000029517 | 100 | (A) HM | (B) Placebo | China | Recruiting | April 30, 2020 | Treatment |
| ChiCTR2000029400 | 60 | (A) HM plus B | (B) Standard care | China | Recruiting | December 31, 2020 | Treatment |
| ChiCTR2000033133 | 60 | (A) HM (Shuanghuanglian) plus B | (B) Standard care | China | Completed | April 20, 2020 | Treatment |
| ChiCTR2000034794 | 60 | (A) HM (Sancai granules) | (B) Standard care | China | Recruiting | April 30, 2020 | Treatment |
| ChiCTR2000036871 | 300 | (A) HM (Jinhua Qinggan granules) | (B) Placebo | China | Recruiting | April 24, 2020 | Treatment |
| ChiCTR2100042066 | 120 | (A) HM (Lianhua Qingwen capsules) | (B No treatment | China | Recruiting | March 31, 2021 | Treatment |
| ChiCTR2100042068 | 20 | (A) HM (Lian-Hua Qing-Ke tablets) plus B | (B) Standard care | China | Recruiting | March 31, 2021 | Treatment |
| ChiCTR2100042069 | 120 | (A) HM (Lian-Hua Qing-Ke tablets) plus B | (B) Standard care | China | Recruiting | March 31, 2021 | Treatment |
| ChiCTR2100044860 | 80 | (A) HM (Qingfei Paidu granules) | (B) Standard care | China | Pending | December 31, 2021 | Treatment |
| ChiCTR2100049663 | 100 | (A) HM (Fuzheng detoxification granules) plus B | (B) Standard care | China | Pending | December 31, 2021 | Treatment |
| ChiCTR2100043012 | 2200 | (A) HM (Lianhua Qingwen capsules) | (B) No intervention | China | Pending | December 1, 2021 | Prevention |
| ChiCTR2200056727 | 860 | (A) HM (Lianhua Qingwen capsules) plus Standard care | (B) Placebo plus Standard care | China | Pending | December 1, 2022 | Treatment |
